# Supplementary material for: Targeted degradation of KRAS and induction of bystander effects by a modular bioPROTAC
Source: Mol Ther Oncol. 2025 Nov 1;33(4):201077. doi: 10.1016/j.omton.2025.201077 (PMC12663003; doi:10.1016/j.omton.2025.201077)
Supplement: Document S1. Figures S1–S6 and Tables S1 and S2 [file mmc1.pdf]

OMTON, Volume *33*

Supplemental information

Targeted degradation of KRAS and induction of bystander effects by a modular bioPROTAC

Shojiro Inano, Akifumi Takaori-Kondo, and Takako Nakajima

Supplemental Information

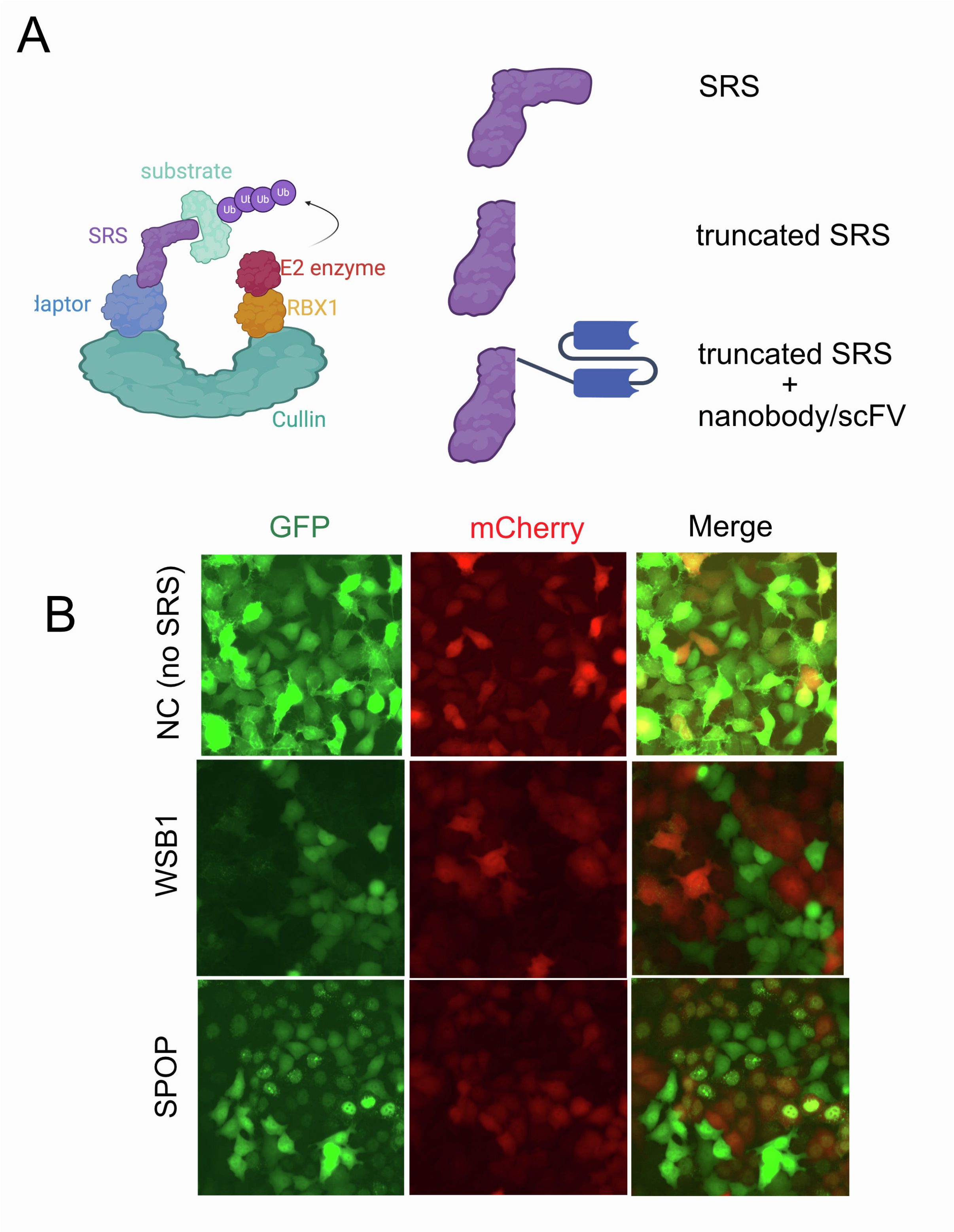

Figure S1. Screening of substrate recognition subunits (SRS) and visualization of EGFP degradation. (A) Schematic of the cullin-RING ligase (CRL) complex and representative substrate recognition subunits (SRS) selected for this study. The SRS consists of an adaptor-binding domain and a target-binding domain. By truncating the native target-binding domain and replacing it with an scFv or VHH, the system can be redirected to degrade arbitrary targets. In this study, a GFP nanobody was used as the target-binding module. (B) Fluorescence microscopy showing EGFP signal reduction in 293T cells expressing HiBiT–EGFP–KRAS and GFP degraders with various SRSs (labeled with mCherry).

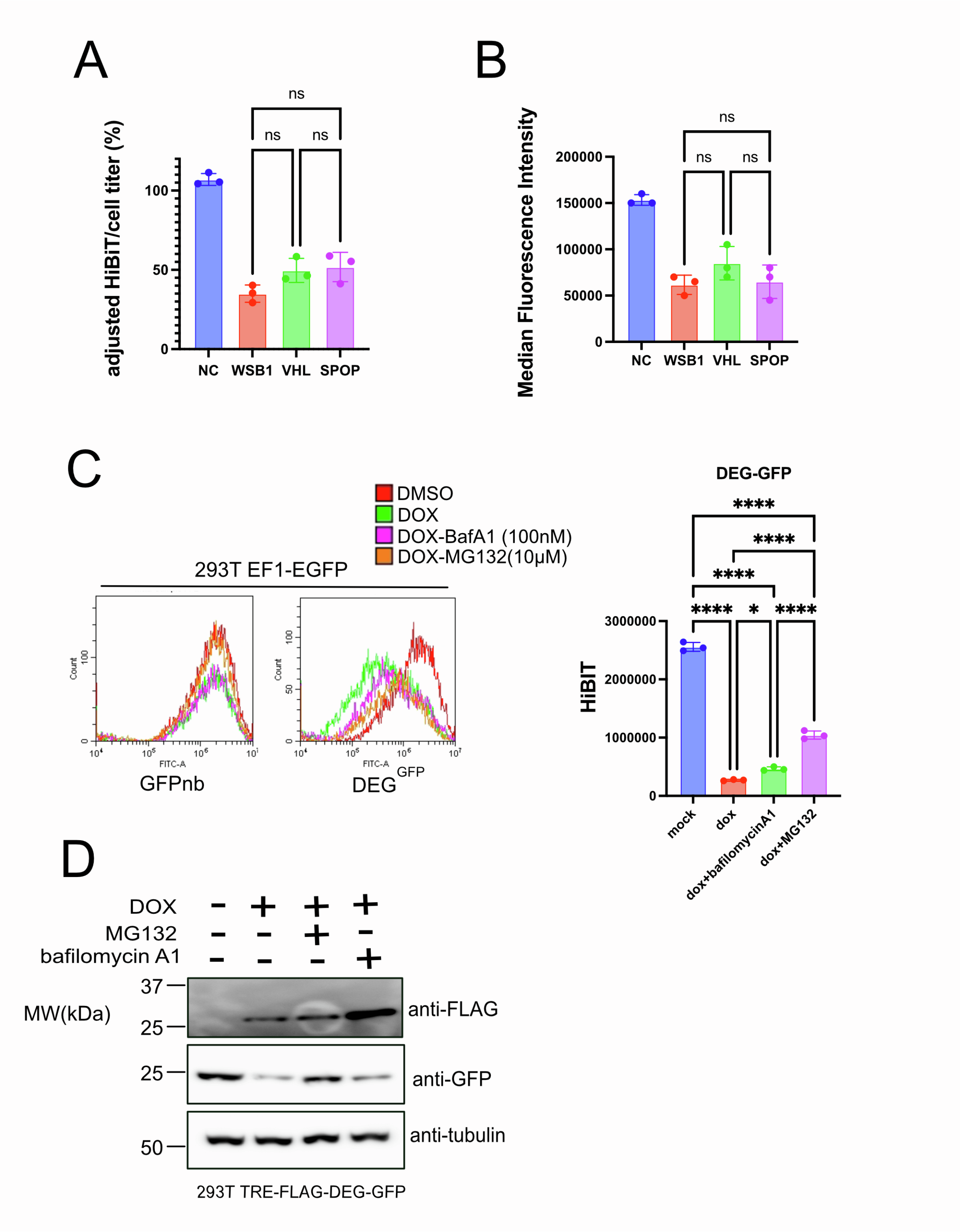

A
adjusted HiBiT/cell titer (%)
ns
NC
WSB1
VHL
SPOP
B
Median Fluorescence Intensity
C
DMSO
DOX
DOX-BafA1 (100nM)
DOX-MG132(10μM)
293T EF1-EGFP
GFPnb
DEG GFP
DEG-GFP
HiBIT
mock
dox
dox+bafilomycinA1
dox+MG132
D
DOX
MG132
bafilomycin A1
MW(kDa)
anti-FLAG
anti-GFP
anti-tubulin
293T TRE-FLAG-DEG-GFP

Figure S2. Functional characterization of SRS constructs and degradation pathways.

(A, B) Flow cytometry (A) and HiBiT assays (B) showing HiBiT-EGFP degradation in 293T cells expressing GFP nanobody–sCD9-SRS proteins (WSB1, VHL, SPOP). No statistically significant differences in degradation efficiency were observed between the constructs, although WSB1 showed a trend toward higher efficiency. (C) Both bafilomycin A1 and MG132 partially inhibited the degradation of DEG-GFP (GFPnanobody-sCD9-WSB1), with MG132 showing a significantly stronger inhibitory effect. Experiments were performed in triplicate, and representative flow cytometry plots along with the median ± SD are shown. (D) Cells in (C) are collected analyzed by immunoblotting. Statistical analysis was performed using one-way ANOVA to compare differences between groups. Data are presented as mean ± SD of three independent biological replicates. Statistical analysis was performed using one-way ANOVA. Data are presented as mean ± SD. $p < 0.05$ (*), $p < 0.01$ (**), $p < 0.001$ (***), $p < 0.0001$ (****).

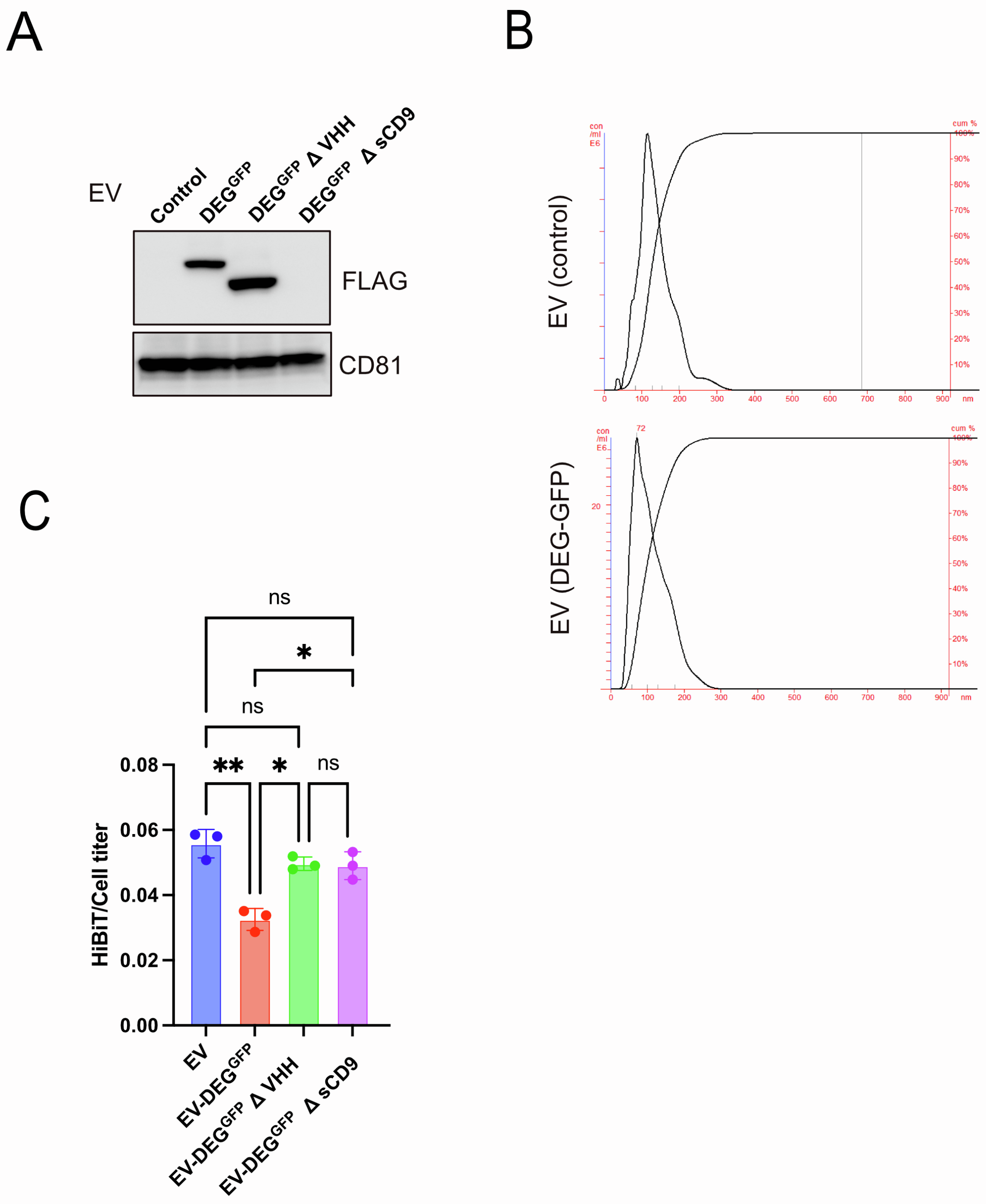
A
B
C
EV
Control
DEGGFP
DEGGFP Δ VHH
DEGGFP Δ sCD9
FLAG
CD81
EV (control)
EV (DEG-GFP)
ns
*
ns
**
*
ns
HiBiT/Cell titer
0.08
0.06
0.04
0.02
0.00
EV
EV-DEGGFP
EV-DEGGFP Δ VHH
EV-DEGGFP Δ sCD9

Figure S3. EV loading of DEG-GFP constructs and functional activity.

(A) Western blot analysis of EVs purified from 293T cells transiently expressing full-length DEG-GFP or deletion mutants lacking the GFP nanobody (ΔVHH), sCD9 (ΔsCD9). EV markers (CD81) and degrader constructs were detected. The ΔsCD9 mutant showed minimal EV incorporation, whereas ΔVHH constructs were successfully loaded.

(B) Nanoparticle Tracking Analysis (NTA) of extracellular vesicles (EVs) isolated from culture supernatants of 293T cells transiently transfected with DEG-GFP or a negative-control vector (NC), as described in Methods. NTA, which estimates particle concentration and size distribution by tracking Brownian motion, showed two overlaid profiles corresponding to the NC and DEG-GFP samples, respectively, with nearly identical size distributions and comparable particle concentrations. These data indicate that sCD9-mediated cargo loading does not substantially alter EV production. Shown is one representative result from two independent experiments.

(C) Recipient 293T cells expressing HiBiT–EGFP–KRAS were treated with the EVs prepared in (A). 5μg of EVs were added per 1 x 10^5 cells. Only EVs containing full-length DEG-GFP induced a significant reduction in HiBiT signal. EVs derived from ΔVHH constructs failed to induce degradation despite successful EV incorporation, while ΔsCD9 EVs lacked effect due to impaired loading. Data are presented as mean ± SD of three independent biological replicates. Statistical analysis was performed using one-way ANOVA. $p < 0.05$ (*), $p < 0.01$ (**), $p < 0.001$ (***), $p < 0.0001$ (****).

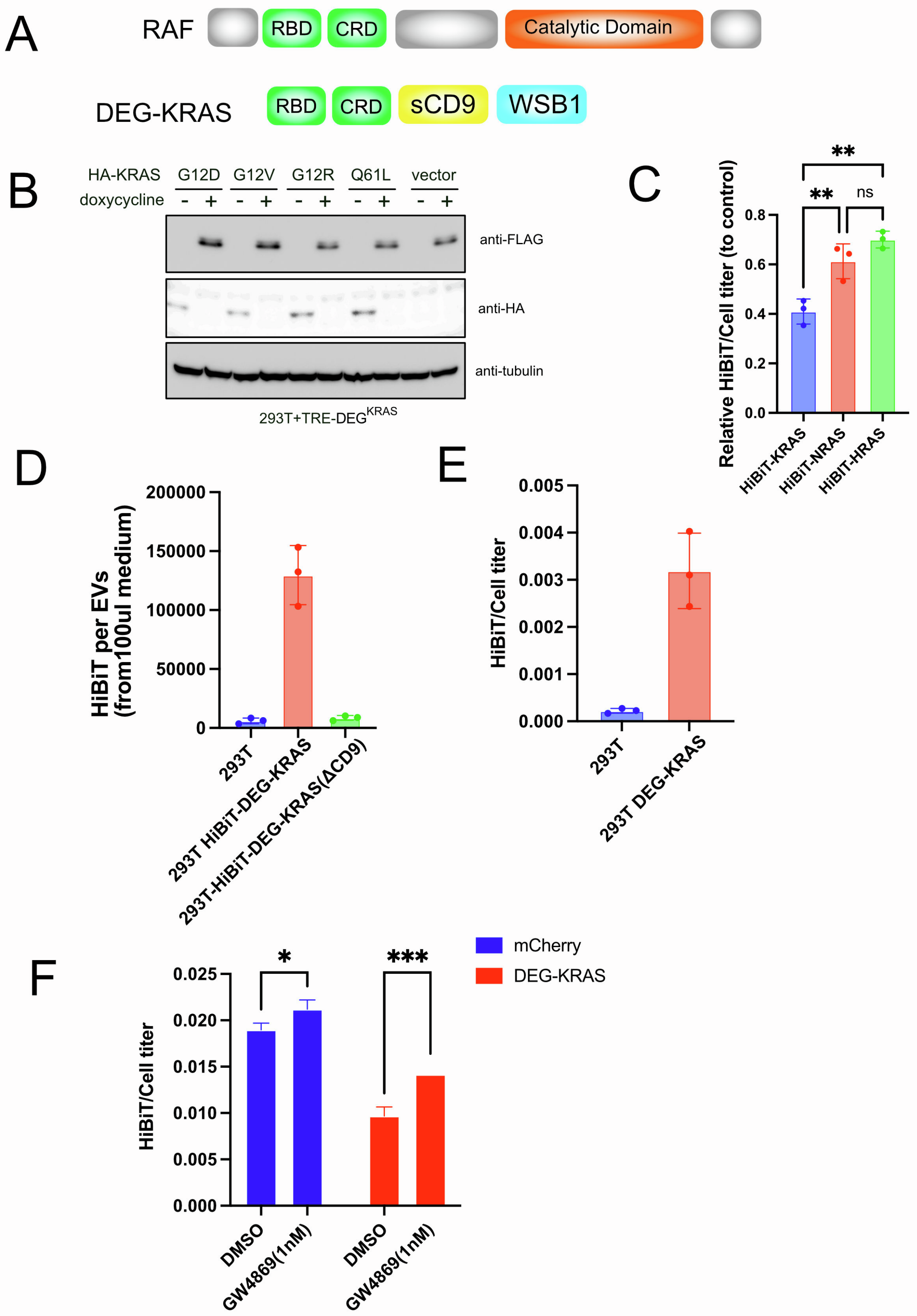
A
RAF
RBD
CRD
Catalytic Domain
DEG-KRAS
RBD
CRD
sCD9
WSB1
B
HA-KRAS
G12D
G12V
G12R
Q61L
vector
doxycycline
- + - + - + - + - +
anti-FLAG
anti-HA
anti-tubulin
293T+TRE-DEG KRAS
C
Relative HiBiT/Cell titer (to control)
**
**
ns
HiBiT-KRAS
HiBiT-NRAS
HiBiT-HRAS
D
HiBiT per EVs (from100ul medium)
293T
293T HiBiT-DEG-KRAS
293T-HiBiT-DEG-KRAS(ΔCD9)
E
HiBiT/Cell titer
293T
293T DEG-KRAS
F
HiBiT/Cell titer
*
***
mCherry
DEG-KRAS
DMSO
GW4869(1nM)
DMSO
GW4869(1nM)

Figure S4. EV-mediated transfer of DEG-KRAS.

(A) Domain organization of CRAF, including the Ras-binding domain (RBD) and cysteine-rich domain (CRD), used to construct DEG-KRAS.

(B) Immunoblot analysis showing KRAS degradation by DEG-KRAS across multiple KRAS mutant variants.

(C) 293T cells stably expressing HiBiT-tagged KRAS, NRAS, or HRAS were treated with doxycycline (1 μg/mL) for 24 hours to induce expression of DEG-KRAS. HiBiT luminescence was measured and normalized to cell viability (CellTiter-Glo). Bar graphs show the relative HiBiT/Cell Titer values compared to no doxycycline control.

(D) 293T–DEG-KRAS donor cells were co-cultured with iRFP-labeled recipient 293T cells in the presence or absence of the EV release inhibitor GW4869. The bystander effect, measured as reduction of HiBiT-tagged KRAS in recipient cells, was attenuated upon GW4869 treatment.

(E) 293T cells stably expressing HiBiT-tagged DEG-KRAS were cultured, and extracellular vesicles (EVs) were purified from conditioned medium. HiBiT activity was measured in the EV fraction, demonstrating secretion of DEG-KRAS via EVs.

(F) 293T donor cells stably expressing HiBiT-tagged DEG-KRAS were co-cultured with iRFP-labeled 293T recipient cells. After co-culture, recipient cells were isolated by flow cytometric sorting of the iRFP-positive population, and HiBiT activity was measured and normalized to cell titer. A small but detectable HiBiT signal was observed in recipient cells, confirming intercellular transfer of DEG-KRAS.

Data are presented as mean ± SD of three independent biological replicates. Statistical analysis was performed using one-way ANOVA. $p < 0.05$ (*), $p < 0.01$ (**), $p < 0.001$ (***), $p < 0.0001$ (****).

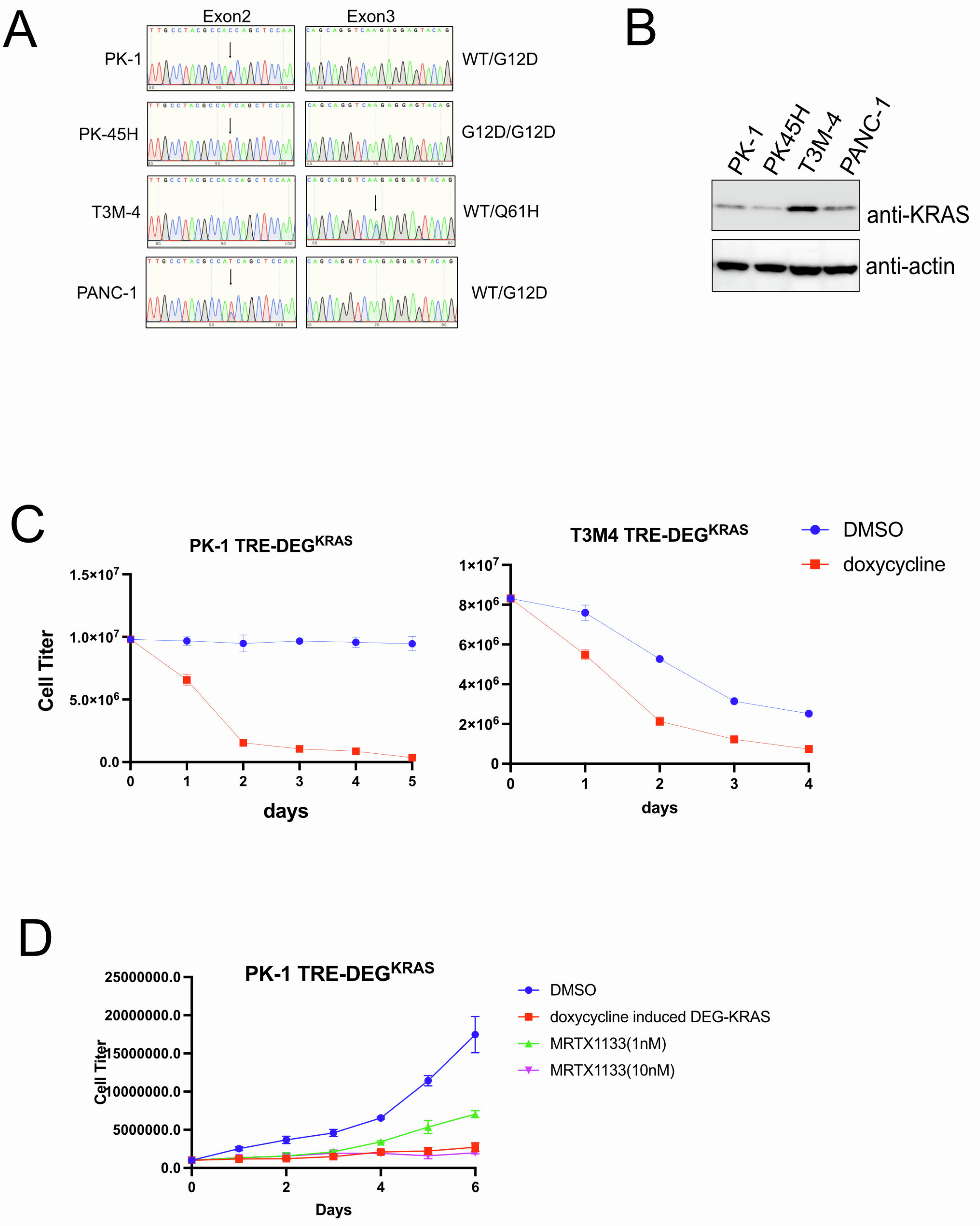
A
Exon2
Exon3
PK-1
WT/G12D
PK-45H
G12D/G12D
T3M-4
WT/Q61H
PANC-1
WT/G12D
B
PK-1
PK45H
T3M-4
PANC-1
anti-KRAS
anti-actin
C
PK-1 TRE-DEG^KRAS
Cell Titer
1.5×10^7
1.0×10^7
5.0×10^6
0.0
0
1
2
3
4
5
days
T3M4 TRE-DEG^KRAS
1×10^7
8×10^6
6×10^6
4×10^6
2×10^6
0
0
1
2
3
4
days
DMSO
doxycycline
D
PK-1 TRE-DEG^KRAS
Cell Titer
25000000.0
20000000.0
15000000.0
10000000.0
5000000.0
0.0
0
2
4
6
Days
DMSO
doxycycline induced DEG-KRAS
MRTX1133(1nM)
MRTX1133(10nM)

Figure S5. Additional characterization of KRAS dependency and DEG-KRAS effects.

(A) KRAS mutation status of five pancreatic cancer cell lines determined by Sanger sequencing.

(B) Baseline KRAS protein expression across the cell lines assessed by immunoblotting.

(C) Cell proliferation of PK-1 and T3M-4 cells expressing DEG-KRAS under suspension (non-adherent) culture conditions.

(D) Comparison of the inhibitory effects of MRTX1133 (KRAS G12D inhibitor) and doxycycline-induced DEG-KRAS expression in PK-1 cells.

Data are presented as mean ± SD of three independent biological replicates.

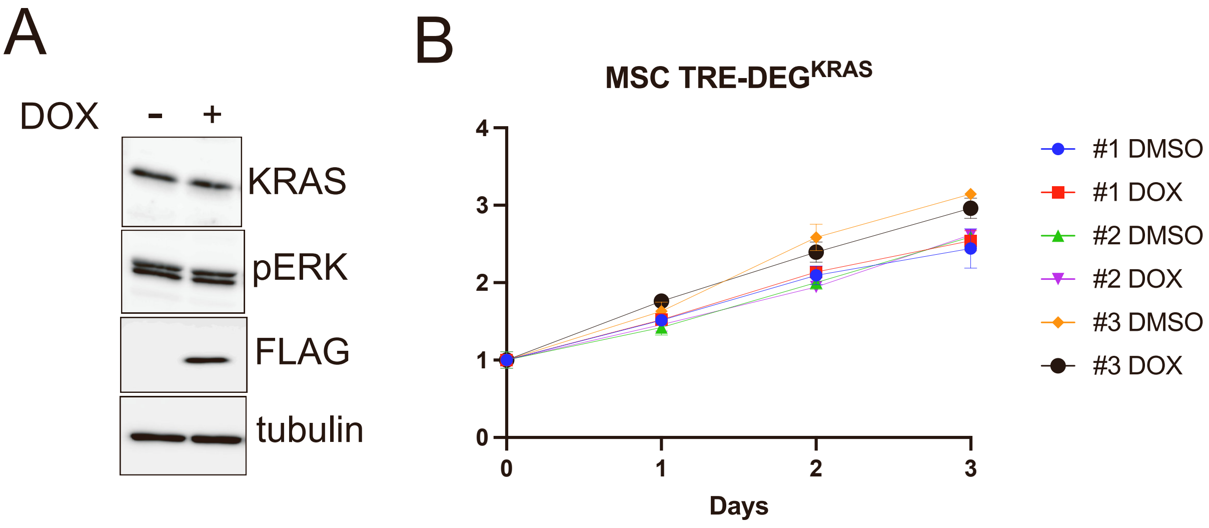

FIgure S6

(A) Western blot confirmation of DEG-KRAS expression in doxycycline-induced MSCs, but KRAS degradation or pERK suppression was not observed.

(B) Proliferation of MSCs was unaffected by DEG-KRAS expression.

Data are presented as mean ± SD of three independent biological replicates.

Table S1

Proteins tested in this paper were listed.

number	Gene	Substrate Recognision	partner Cullin	synonyms	length (a.a)
1	FBXL1	C-teminus	CUL1	SKP2	424
2	FBXO6	C-teminus	CUL1	FBX6	293
3	FBXO21	C-teminus	CUL1/CUL3	FBX21	628
4	FBXO27	C-teminus	CUL1	FBX27	283
5	FBXO41	C-teminus	CUL7	FBX41	875
6	FBW1B	C-teminus	CUL1	FBXW11	542
7	FBXW5	C-teminus	CUL7/CUL3	FBW5	566
8	FBXW9	C-teminus	CUL7	FBW9	458
9	FBXW12	C-teminus	CUL1	FBW12	464
10	WSB1	N-terminus	CUL5/CUL2	SWIP1	421
11	VHL	N-terminus	CUL2,/CUL3/CUL5	proteinG7	213
12	CRBN	C-teminus	CUL4		442
13	SOCS1	N-terminus	CUL2	SSI1, TIP3	211
14	SPOP	N-terminus	CuUL3	HIB homolog 1	374

Table S2

Antibodies used in this study were listed.

antigen	host	supplier	Cat #
GFP	rabbit	proteintech	66301-1-Ig
FLAG	mouse	Wako	014-27763
SPOT	alpaca	proteintech	ebAF488
KRAS	rabbit	thermo	12063-1-AP
ERK	mouse	SCBT	sc-514302
p-ERK	mouse	SCBT	sc-81492
HA	rabbit	proteintech	51064-2-AP
tubulin	mouse	Wako	013-25033
p-Akt	mouse	proteintech	66444-1-Ig
RhoA	mouse	SCBT	sc-418
actin	rabbit	proteintech	23660-1-AP
