# Supplementary material for: Targeted degradation of KRAS and induction of bystander effects by a modular bioPROTAC
Source: Mol Ther Oncol. 2025 Nov 1;33(4):201077. doi: 10.1016/j.omton.2025.201077 (PMC12663003; doi:10.1016/j.omton.2025.201077)
Supplement: Document S2. Article plus supplemental information [file mmc2.pdf]

Molecular Therapy
Oncology
Original Article

Targeted degradation of KRAS and induction of bystander effects by a modular bioPROTAC

Shojiro Inano,[1,3] Akifumi Takaori-Kondo,[2] and Takako Nakajima[1]

[1]Department of Early Clinical Development, Graduate School of Medicine, Kyoto University, Yoshida-Konoecho, Sakyo-ku, Kyoto 606-8501, Japan; [2]Department of Hematology, Graduate School of Medicine, Kyoto University, Yoshida-Konoecho, Sakyo-ku, Kyoto 606-8501, Japan; [3]Department of Medical Research, Medical Research Institute Tazuke-Kofukai Kitano Hospital, 2-4-20, Ougimachi, Kita-ku, Osaka, Japan

Targeted protein degradation is a promising strategy for addressing oncogenic drivers that are difficult to inhibit with small molecules, such as KRAS. While bioPROTACs expand the range of targetable proteins, their clinical translation is limited by inefficient delivery. To overcome this barrier, we engineered a chimeric protein, termed DEG-KRAS, which consists of a KRAS-binding domain derived from CRAF (RBD/CRD), an E3 adaptor (WSB1), and an optional trafficking module. DEG-KRAS induced degradation of active KRAS and suppressed proliferation in pancreatic cancer cell lines by reducing phospho-ERK levels. Notably, DEG-KRAS expression in mesenchymal stem cells (MSCs) exerted a bystander effect, leading to KRAS degradation and growth inhibition in co-cultured cancer cells. Specificity was confirmed using control constructs lacking each functional domain. Although the antiproliferative effect was modest compared to direct expression in cancer cells, the indirect impact highlights a non-cell-autonomous mechanism. While the precise mode of intercellular transfer remains to be elucidated, these findings suggest the involvement of extracellular vehicles or other secretory pathways. This strategy may offer a novel therapeutic avenue for targeting KRAS-driven tumors, particularly pancreatic adenocarcinoma.

INTRODUCTION

Advances in molecular therapeutics have transformed the landscape of cancer treatment. Although prognosis has improved in many cancers and durable remissions have become possible in selected cases, a substantial proportion of patients still face poor outcomes. This underscores the need for continued development of novel therapeutic strategies.

Molecularly targeted therapies can be broadly divided into two categories: those directed at extracellular targets, such as monoclonal antibodies and chimeric antigen receptor T (CAR-T) cells, and those aimed at intracellular targets, typically using small-molecule inhibitors. While small molecules have successfully modulated several oncogenic drivers such as kinases[1] and epigenetic regulators such as BET,[2,3] they remain ineffective against many intracellular proteins due to structural constraints or the lack of suitable binding pockets. This has led to the rise of targeted protein degradation strategies, including PROTACs (proteolysis-targeting chimeras), which eliminate target proteins by recruiting E3 ligases for ubiquitination and subsequent proteasomal degradation.[4]

Although PROTACs have broadened the scope of druggable targets, they still depend on small-molecule binding to the target protein, limiting their applicability to proteins with well-defined and accessible pockets. To address this, bioPROTACs have been developed as protein-based degraders that use antibody fragments or nanobodies for substrate recognition, coupled to E3 ligase components.[5] These systems offer greater flexibility in target selection, including proteins that are structurally intractable to small molecules. However, bioPROTACs face a major challenge: they cannot penetrate cell membranes and therefore require efficient delivery platforms, such as viral vectors or nanoparticles, which are often limited by tissue specificity, immunogenicity, or gene transfer efficiency.

To circumvent these limitations, we sought to develop a bioPROTAC-based system capable of acting beyond the cells in which it is expressed, thereby enabling non-cell-autonomous degradation of target proteins. Specifically, we hypothesized that extracellular vesicles (EVs) could serve as a delivery vehicle to transfer bioPROTACs to neighboring cells. EVs are lipid bilayer-enclosed particles naturally secreted by cells and are known to carry functional proteins, RNAs, and signaling molecules across cell populations.[6] Their ability to mediate intercellular communication, combined with their compatibility with endogenous trafficking pathways, makes them a promising tool for delivering therapeutic proteins.

In this study, we designed a modular chimeric protein termed DEG-KRAS, composed of a KRAS-binding domain derived from CRAF, an EV-trafficking domain (short CD9 [sCD9]), and an E3 adaptor subunit (WSB1). This construct was engineered to both induce degradation of KRAS within expressing cells and exert a bystander effect on adjacent cells through EV-mediated transfer. Using this system, we demonstrated selective degradation of activated KRAS in

Received 29 July 2025; accepted 29 October 2025;
https://doi.org/10.1016/j.omton.2025.201077.

Correspondence: Shojiro Inano, Department of Early Clinical Development, Graduate School of Medicine, Kyoto University, Yoshida-Konoecho, Sakyo-ku, Kyoto 606-8501, Japan.
E-mail: shoin@kuhp.kyoto-u.ac.jp

© 2025 The Author(s).
Published by Elsevier Inc. on behalf of The American Society of Gene and Cell Therapy.
This is an open access article under the CC BY license (http://creativecommons.org/licenses/by/4.0/).

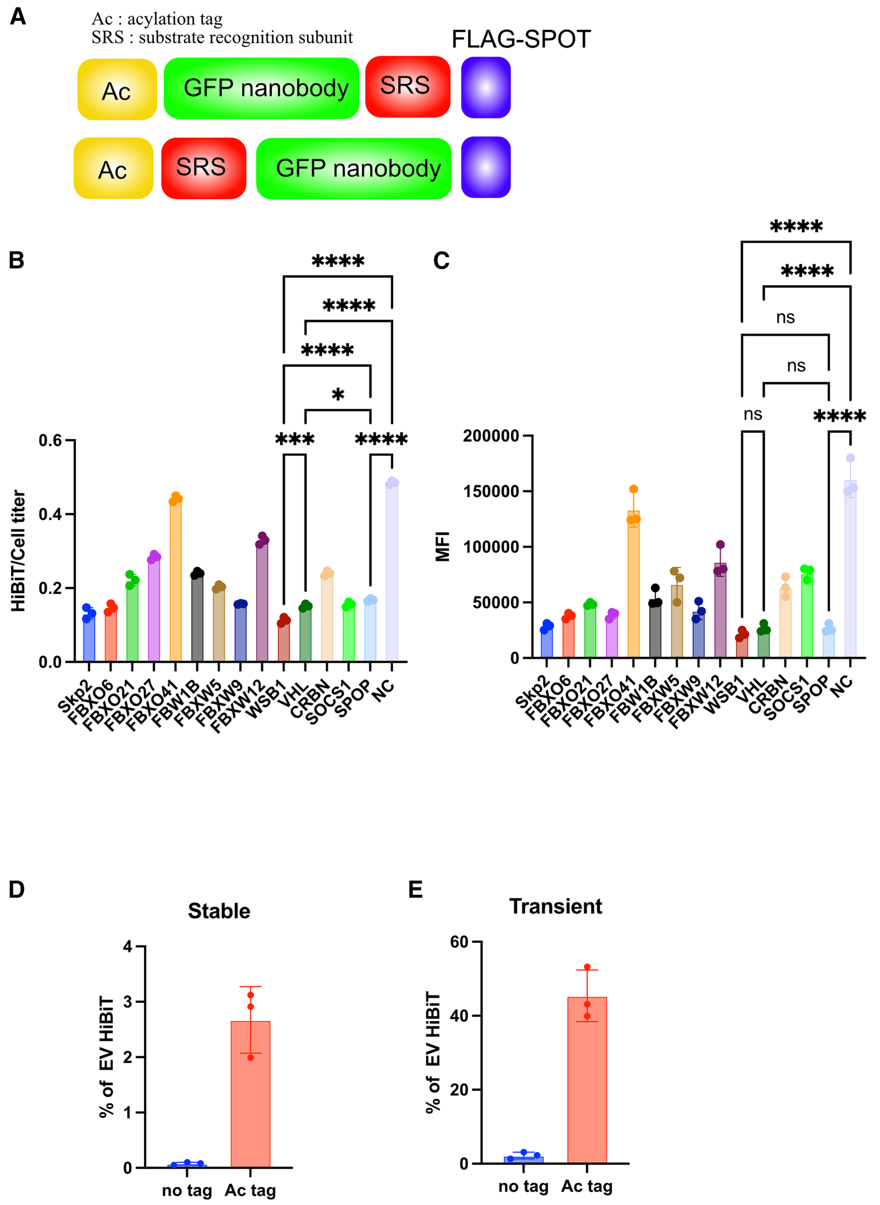

Figure 1. Design of a chimeric protein enabling both EV incorporation and target degradation

(A) Schematic of constructs comprising an acylation tag (Ac), an SRS, and a FLAG-SPOT tag. (B) HiBiT/CellTiter ratios in 293T cells expressing HiBiT-tagged EGFP with different SRS constructs after doxycycline induction. (C) Flow cytometry analysis of EGFP median fluorescence intensity (MFI) in the same cells as in (B). (D) HiBiT signals in culture supernatants and cell lysates of stable 293T cells expressing GFP nanobody-WSB1-HiBiT with or without the Ac tag. (E) HiBiT signals in culture supernatants and cell lysates of transiently transfected 293T cells expressing the same constructs as in (D). Data are presented as mean ± SD of three independent biological replicates. Statistical analysis was performed using one-way ANOVA. **p* < 0.05, ****p* < 0.001, *****p* < 0.0001.

multiple pancreatic cancer cell lines, along with suppression of downstream ERK signaling and proliferation.

To explore potential clinical applications, we also engineered mesenchymal stem cells (MSCs) to express DEG-KRAS, leveraging their natural ability to secrete EVs and function as home for tumor microenvironments.[7–9] While the antiproliferative effect was modest compared to direct expression of the construct in cancer cells, MSC-mediated delivery resulted in significant suppression of tumor cell growth in co-culture systems.

These findings establish a proof of concept for EV-based delivery of bioPROTACs and suggest that this approach may serve as a foundation for future strategies targeting proteins that are otherwise difficult to inhibit.

RESULTS

Creation of a protein that achieves simultaneous exosome translocation and GFP degradation

To enable diffusion of bioPROTAC activity beyond the originating cells, we focused on engineering constructs that are trafficked into EVs. EVs are membrane-bound particles released into the extracellular space and are known to mediate intercellular communication by transporting proteins, lipids, and nucleic acids.[10,11] We, therefore, designed a chimeric protein that combines three essential modules: an EV trafficking domain, a substrate recognition domain, and an E3 ligase adaptor domain (Figure 1A).

As the EV trafficking domain, we initially selected an acylation tag based on its short sequence and prior evidence that lipid modifications can facilitate vesicular membrane association.[12] For substrate recognition, we adopted a nanobody scaffold, which is derived from camelid heavy-chain antibodies and has been widely used in bioPROTAC systems due to its small size (~15 kDa), high stability, and target specificity.[13–15] To validate the system, we utilized a nanobody specific for GFP, allowing for quantitative readout of target degradation.[16]

Among the over 600 known E3 ligases,[17] we focused on members of the cullin-RING ligase (CRL) family, given their compact modularity and compatibility with EV loading. CRLs typically consist of a scaffold cullin protein, a RING domain, and a substrate recognition subunit (SRS), of which the SRS provides specificity for

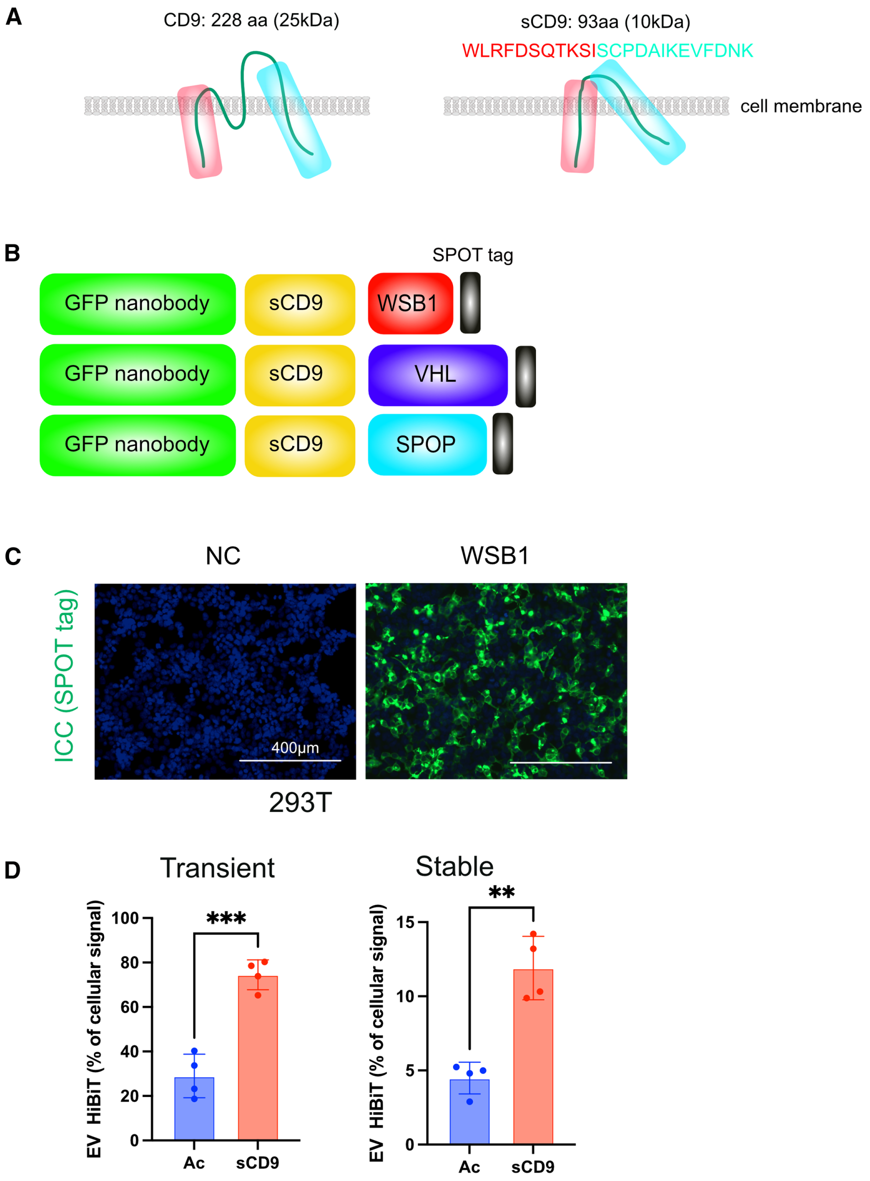

Figure 2. Optimization of the EV-trafficking domain using short CD9

(A) Schematic comparison of full-length CD9 and truncated sCD9 used as EV-targeting domains. (B) Domain architecture of the chimeric protein DEG-GFP using sCD9 as the trafficking tag. To enable detection, a SPOT tag (amino acid sequence: PDRVRAVSHWSS) was fused to the C terminus of each construct. (C) 293T cells transiently expressing SPOT-tagged DEG-GFP (WSB1) were analyzed by immunocytochemistry. (D) HiBiT-tagged constructs (Ac-GFPnb-WSB1 or GFPnb-sCD9-WSB1) were transiently (left) or stably (right) expressed in 293T cells. After 24 h, HiBiT signals in cell lysates and supernatants were separately measured, and the EV-to-intracellular HiBiT ratio was calculated. Data are presented as mean ± SD of three independent biological replicates. Statistical analysis was performed using one-way ANOVA. $^{**}p < 0.01$, $^{***}p < 0.001$.

degradation of targets (Figure S1A). To minimize construct size while maintaining function, we selected SRSs known to interact with cullin families, which have numerous characterized binding partners.

We generated GFP-degrading chimeric constructs by replacing the target-binding region of each SRS with a GFP nanobody, while retaining the adaptor interaction domains (Figure S1A; Table S1). These constructs were expressed in a doxycycline-inducible manner in 293T cells stably expressing HiBiT-EGFP reporter. Target degradation was assessed by both flow cytometry and HiBiT assays. The HiBiT system, developed by Promega, utilizes an 11-amino-acid tag that forms a luminescent complex upon association with LgBiT, allowing quantitative measurement of tagged proteins. Among the constructs tested, those incorporating WSB1, VHL, or SPOP showed the strongest degradation effects in both assays (Figures 1B and 1C). Fluorescence microscopy further confirmed the loss of EGFP signal upon degrader induction (Figure S1B).

To assess vesicle trafficking of the GFP degrader protein, a HiBiT tag was fused to the C terminus (GFPnanobody-WSB1-HiBiT with or without Ac tag) and expressed stably in 293T cells. HiBiT signal in the culture supernatant was compared with that in whole-cell lysates. This analysis revealed that approximately 2%–3% of the expressed degrader protein was recovered in the extracellular fraction when an Ac tag was appended (Figure 1D). Notably, transient overexpression markedly increased the amount of degrader protein associated with EVs, indicating that the expression level is a critical determinant of EV loading efficiency (Figure 1E).

Conversion into a sCD9 tag with better EV transition

Although the acylation tag used in the initial constructs is compact and minimally disruptive to protein function, its efficiency for promoting EV incorporation was relatively low. To improve EV trafficking, we turned to CD9, a tetraspanin protein abundantly enriched in exosomes. However, full-length CD9 is a multifunctional membrane protein[18,19] and its overexpression could interfere with normal cellular signaling. To mitigate this concern, we designed a modified version of its extracellular domain, termed short CD9 (sCD9), which retains vesicular trafficking properties, while minimizing potential off-target effects (Figure 2A).[20]

We constructed sCD9-fused versions of the previously tested degraders containing WSB1, VHL, and SPOP as E3 adaptor domains (Figure 2B). These chimeric proteins retained GFP degradation capability, as assessed by HiBiT assays and flow cytometry (Figures S2A and S2B). Given its compact size and consistent performance, WSB1 was selected as the E3 module for downstream applications. The final construct, referred to as DEG-GFP (GFP nanobody-sCD9-WSB1), served as the prototype for further analysis.

To confirm the degradation mechanism of DEG-GFP, we examined the effect of a proteasome inhibitor on its stability. Proteasome inhibition with MG132 substantially rescued EGFP levels in DEG-GFP-expressing cells, confirming that the degradation was mediated by the ubiquitin-proteasome system and not due to passive release or competition. A slight increase in EGFP was also observed upon treatment with bafilomycin A1, suggesting a minor contribution of lysosomal degradation; however, the proteasome appeared to play the dominant role (Figures S2C and S2D). In addition to its degradation profile, we next examined the subcellular localization and EV-loading efficiency of DEG-GFP. Immunocytochemistry revealed a membrane-associated distribution of DEG-GFP, with additional punctate endosomal and diffuse cytoplasmic signals, consistent with sCD9-mediated trafficking through both plasma membrane and endosomal compartments (Figure 2C). Furthermore, HiBiT-tagged DEG-GFP demonstrated significantly higher incorporation into the EV fraction compared to the acylation-tagged counterpart, confirming the superior EV-loading efficiency of the sCD9 domain (Figure 2D).

Degradation of EGFP-KRAS via bystander effects in co-culture

To exert a functional effect in recipient cells, bioPROTACs delivered via EVs must escape the endosomal compartment and reach the cytosol.[20,21] Given that the sCD9 tag confers membrane association (Figure 2C), we reasoned that membrane-localized proteins would be ideal targets for EV-mediated degradation.

KRAS was selected as a model target due to its clinical relevance and notorious resistance to small-molecule inhibition. KRAS mutations resulting in constitutive activation are commonly found in pancreatic, colorectal, and lung cancers.[22–24] Although the dependence of pancreatic cancer cells on KRAS has been debated,[16–19] sustained KRAS activity is generally considered essential for tumor maintenance, making it a compelling therapeutic target.

We first established 293T cells stably expressing HiBiT-tagged EGFP-KRAS fusion protein. To test EV-mediated delivery of degrader constructs, we purified EVs from the culture supernatant of 293T cells transiently overexpressing full-length DEG-GFP or deletion variants lacking one of the three functional domains: the GFP nanobody (ΔVHH), and sCD9 (ΔsCD9). Western blotting confirmed the presence of EV markers and efficient incorporation of DEG-GFP, ΔVHH, while the ΔsCD9 construct was barely detectable, indicating that sCD9 is essential for EV loading (Figure S3A). Notably, nanoparticle tracking analysis, which estimates vesicle concentration and size distribution by tracking the Brownian motion of individual particles, revealed no significant differences in either particle number or size distribution upon sCD9 overexpression. The two profiles shown in Figure S3B represent control and sCD9-overexpressing samples/two independent replicates, both exhibiting nearly overlapping distributions. These data indicate that the overall production and release of EVs were not substantially altered, supporting the conclusion that sCD9 primarily enhances cargo loading rather than affecting EV biogenesis. (Figure S3B).

These EV preparations were then added to recipient 293T cells expressing HiBiT-EGFP-KRAS. EVs containing full-length DEG-GFP induced a significant reduction in HiBiT signal, indicative of KRAS degradation. In contrast, EVs from ΔVHH and ΔsCD9 mutants failed to induce degradation, denying the possibility of nonspecific effect (Figure S3C).

To further validate these findings under more physiological conditions, we leveraged the previously reported SLEEQ assay (single-step labeling of EVs with enhanced quantification). This method enables the quantitative detection of EV-incorporated proteins in culture supernatants by tagging the protein of interest with a HiBiT peptide, which complements with LgBiT added to the extracellular medium to generate luminescence. Because the luminescence signal only arises when HiBiT-tagged proteins are secreted or packaged into EVs and released extracellularly, SLEEQ allows for sensitive and high-throughput evaluation of EV loading without requiring ultracentrifugation or EV purification.[25] We have previously shown that co-culture with recipient cells yields substantially higher SLEEQ signals than application of purified EVs, indicating more efficient cytoplasmic delivery under co-culture conditions.[20]

Accordingly, 293T cells expressing mCherry-labeled DEG-GFP under doxycycline control were co-cultured with recipient 293T cells stably expressing HiBiT-EGFP-KRAS. Flow cytometry analysis of the mCherry-negative population (corresponding to recipient cells) revealed a marked reduction in EGFP-KRAS expression (Figure 3A), which was further corroborated by fluorescence microscopy showing diminished membrane-associated signal (Figure 3B). Consistently, HiBiT signals normalized to cell titer in the mCherry-negative population were also significantly decreased, further confirming the reduction in recipient cells (Figure 3C). In contrast, co-culture with deletion mutants used in Figure S2C did not result in a significant decrease in HiBiT signal, supporting a bystander degradation mechanism of EGFP-KRAS.

Application to KRAS degradation

Based on the observed ability of DEG-GFP to degrade membrane-associated fusion proteins, we next aimed to target endogenous KRAS using a rationally designed chimeric degrader. KRAS is a small GTPase that functions as a molecular switch by cycling between an active, GTP-bound state and an inactive, GDP-bound state.[26] This binary switching mechanism plays a central role in controlling various intracellular signaling cascades. While nanobodies specific

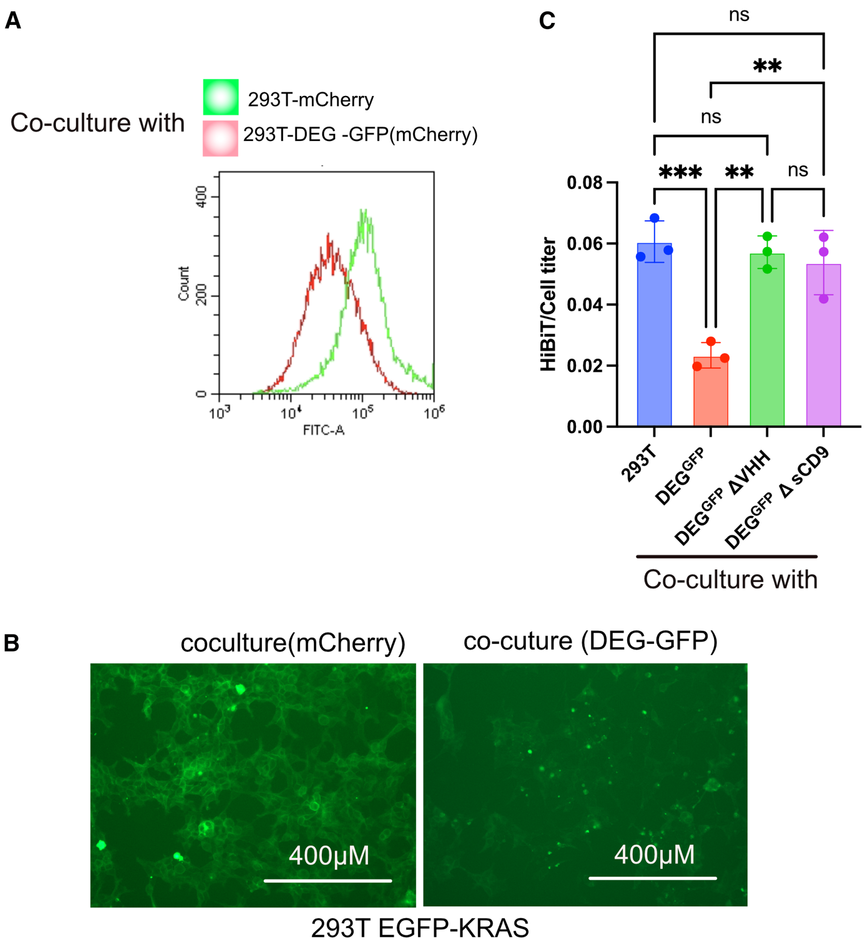

Figure 3. EV-mediated bystander degradation of membrane-bound EGFP-KRAS

(A) mCherry-labeled 293T cells expressing DEG-GFP were co-cultured with recipient 293T cells expressing HiBiT-EGFP-KRAS. After 48 h, EGFP expression was analyzed by flow cytometry in the mCherry-negative gate. (B) Fluorescence microscopy of co-cultured cells showing reduced EGFP signal. (C) HiBiT signals and total CellTiter values were measured from the co-culture samples. The proportion of mCherry-negative recipient cells was determined by flow cytometry and used to estimate the CellTiter value of the recipient population. HiBiT signals were then normalized to the estimated CellTiter value of recipient cells to calculate the HiBiT/CellTiter ratio. Data are presented as mean ± SD of three independent biological replicates. Statistical analysis was performed using one-way ANOVA. $^{**}p < 0.01$, $^{***}p < 0.001$.

to mutant KRAS represent a rational approach, we opted to target the active form of KRAS more broadly to accommodate potential compensation by wild-type alleles.

One of the major effectors of active KRAS is the RAF-MEK-ERK pathway.[27] Among RAF isoforms, both BRAF and CRAF have been well characterized and share a conserved structural organization.[28,29] The Ras-binding domain (RBD) in CRAF mediates direct interaction with KRAS, and the adjacent cysteine-rich domain (CRD) is also thought to contribute to the stability of this interaction.

To exploit this interface, we constructed a new chimeric protein, termed DEG-KRAS, in which the target recognition domain was composed of the RBD and CRD regions from CRAF (Figure S4A). When expressed in 293T cells in a doxycycline-dependent manner, DEG-KRAS reproducibly reduced endogenous KRAS levels across independent experiments. Although epidermal growth factor stimulation tended to further decrease KRAS expression, this effect did not reach statistical significance (Figure 4A). The degradation was fully abrogated by treatment with the proteasome inhibitor MG132, confirming that the mechanism involves the ubiquitin-proteasome system (Figure 4B).

To evaluate the selectivity of DEG-KRAS for the active form of KRAS, we generated 293T cell lines stably expressing hemagglutinin (HA)-tagged KRAS constructs, including the constitutively active mutant KRAS G13D and the inactivated double mutant KRAS G13D/K104Q.[30] DEG-KRAS expression by doxycycline significantly reduced HA-tagged KRAS in cells expressing KRAS G13D, but had only a minor effect on the G13D/K104Q mutant (Figure 4C). Wild-type KRAS showed an intermediate level of degradation, supporting the preferential activity of DEG-KRAS toward the GTP-bound, active conformation. Consistent results were obtained when the HA tag was replaced with a HiBiT tag; cell titer-normalized HiBiT signals showed a similar pattern of differential degradation across the KRAS variants (Figure 4D). To examine whether this effect extends to other active KRAS mutants, we evaluated additional variants (G12D, G12V, G12R, and Q61L). To examine whether this effect extends to other active KRAS mutants, we evaluated additional variants (G12D, G12V, G12R, and Q61L). In all cases, western blot analysis revealed nearly complete degradation of the mutant KRAS proteins upon DEG-KRAS expression, comparable to the results observed with G12C (Figure S4B). To examine whether DEG-KRAS exerts bystander effects, we co-cultured 293T donor cells expressing DEG-KRAS and mCherry with recipient cells expressing HiBiT-tagged KRAS. After 24 h of co-culture, HiBiT and CellTiter signals were measured from the total population. The proportion of recipient cells was estimated based on the frequency of mCherry-negative cells, determined by flow cytometry. Because HiBiT was fused to the target protein, luminescence signals reflected KRAS abundance in recipient cells. By normalizing HiBiT signals to the estimated CellTiter value of the mCherry-negative population, we calculated the amount of target protein per recipient cell. A significant decrease in the HiBiT/CellTiter ratio was observed in the presence of DEG-KRAS-expressing donor

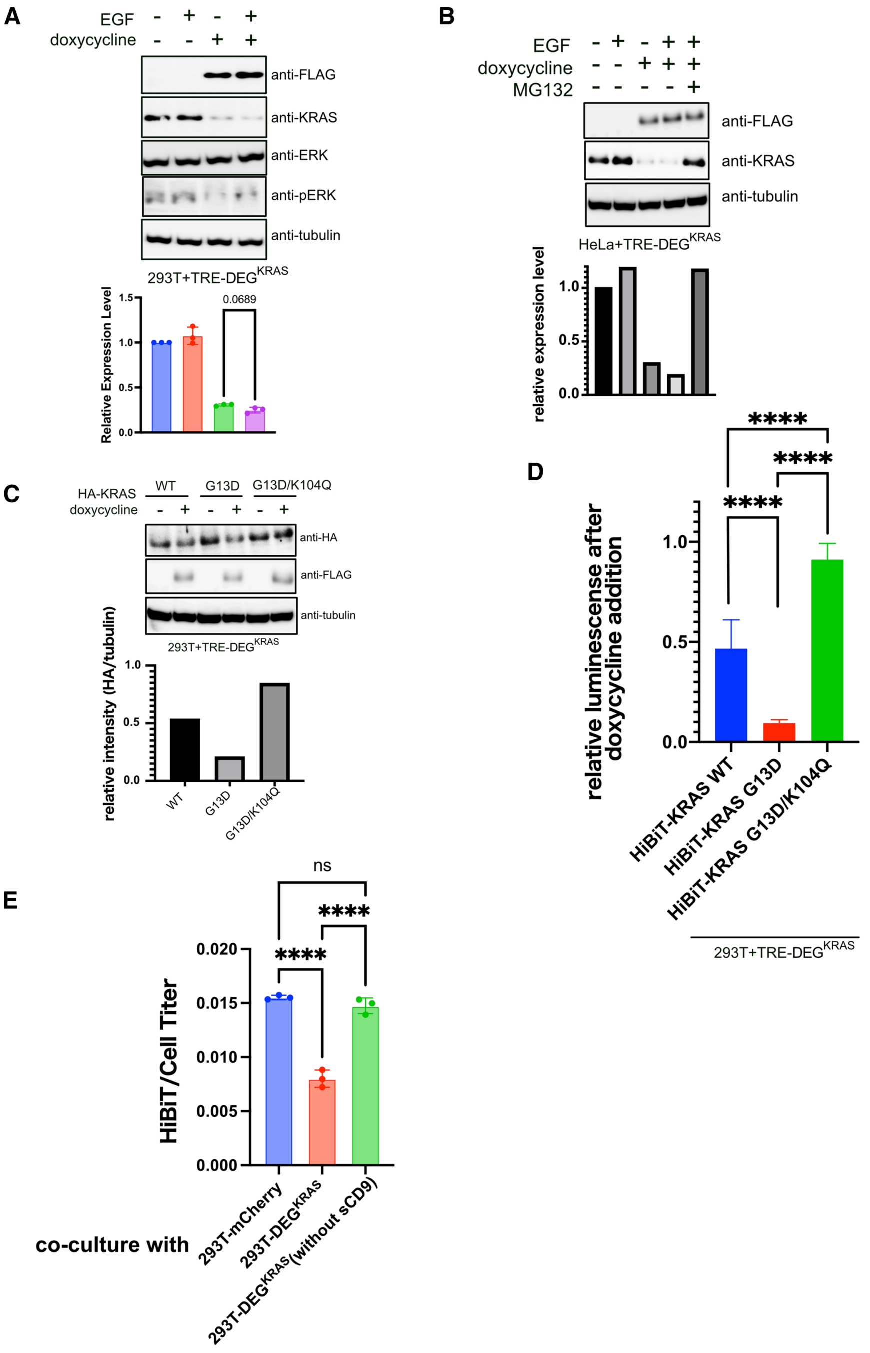

Figure 4. Selective degradation of activated KRAS using DEG-KRAS

(A) Immunoblot analysis of KRAS and phospho-ERK in 293T cells induced with DEG-KRAS in a doxycycline-dependent manner, with or without EGF stimulation. Quantification of KRAS normalized to tubulin is shown. Data are presented as mean ± SD of three independent biological replicates. (B) Immunoblot analysis in HeLa cells, with MG132 treatment where indicated. (C) Immunoblot analysis of 293T cells stably expressing HA-tagged KRAS (WT or mutants) after doxycycline-induced DEG-KRAS expression. (D) HiBiT assay of 293T cells stably expressing HiBiT-tagged KRAS (WT or mutants) after doxycycline-induced DEG-KRAS expression. Data are presented as mean ± SD of three independent biological replicates. (E) Co-culture of DEG-KRAS/mCherry donor cells with recipient cells expressing HiBiT-tagged KRAS. HiBiT signals normalized to CellTiter values of mCherry-negative recipient cells are shown. Data are presented as mean ± SD of three independent biological replicates. Statistical analysis was performed using one-way ANOVA. ****$p < 0.0001$.

cells, suggesting EV-mediated bystander degradation (Figure 4E). In contrast, a version of DEG-KRAS lacking the sCD9 EV-trafficking domain failed to induce bystander degradation, confirming this domain is essential to exert bystander effects.

We then stably expressed HiBiT-tagged versions of KRAS, NRAS, or HRAS in 293T cells and monitored the effects of doxycycline-induced DEG-KRAS expression on HiBiT signal normalized to cell viability (HiBiT/CellTiter). While a moderate decrease in HiBiT/CellTiter was observed for HiBiT-NRAS and HiBiT-HRAS, the reduction was significantly more pronounced in HiBiT-KRAS-expressing cells, indicating preferential degradation of KRAS over the other RAS isoforms (Figure S4C).

Finally, we tried to investigate the mechanism underlying the bystander effect. We first stably expressed HiBiT-tagged DEG-KRAS and detected HiBiT signals in the purified EVs, indicating secretion into EVs (Figure S4D). Next, following co-culture of 293T cells stably expressing HiBiT-tagged DEG-KRAS (donor) with iRFP-labeled 293T cells (recipient), we sorted the iRFP-positive population and measured HiBiT activity normalized to cell titer. This analysis revealed a small but detectable signal in recipient cells, confirming intercellular transfer of DEG-KRAS (Figure S4E). Furthermore, inhibition of EV release with GW4869 reduced the bystander effect (Figure S4F), supporting that EV-mediated transport contributes to this process. Together, these data suggest that EVs are, at least partially, responsible for DEG-KRAS transfer, although the process appears inefficient and may require optimization.

Proliferation inhibition of pancreatic cancer via induction of DEG^{KRAS}

While DEG-KRAS effectively degraded KRAS in 293T and HeLa cells, further validation in disease-relevant models was required.

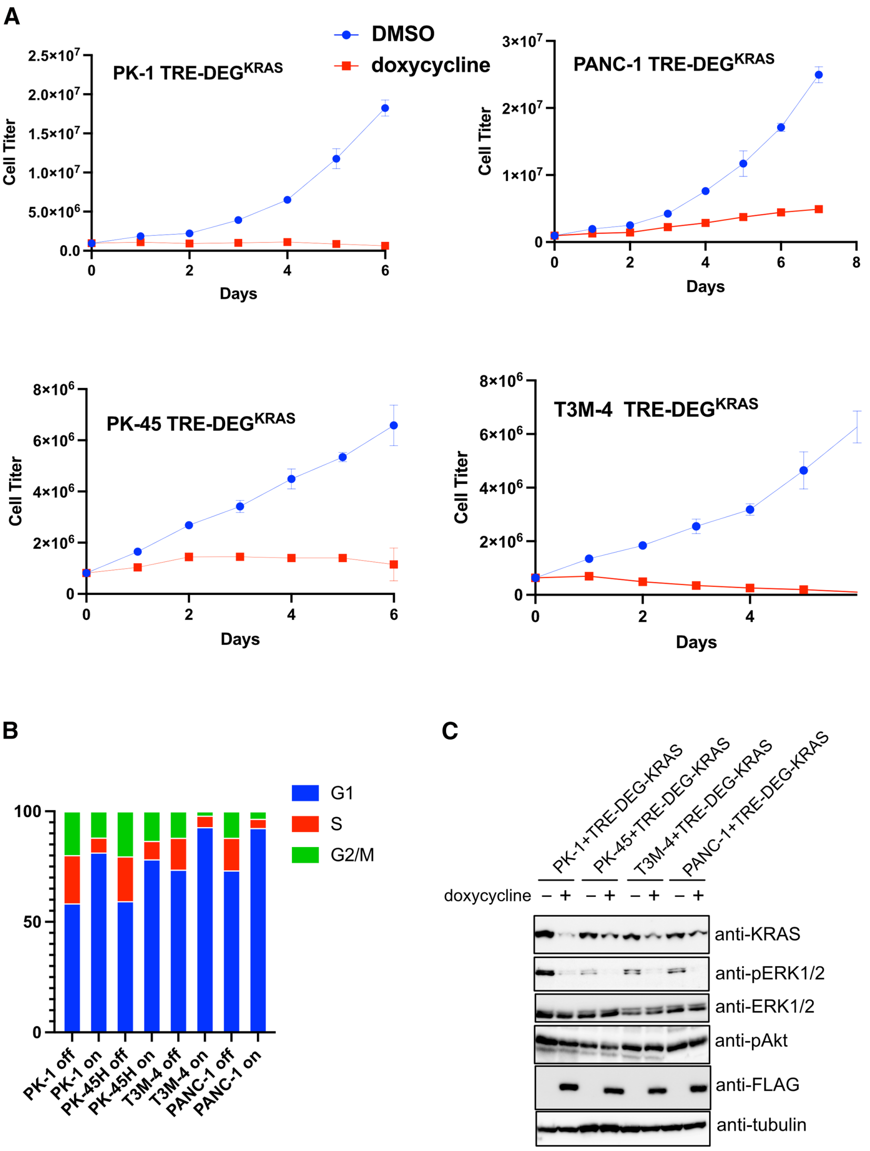

Figure 5. DEG-KRAS inhibits proliferation of pancreatic cancer cells

(A) Cell proliferation assay of PK-1, PK-45H, PANC-1, and T3M-4 cells expressing DEG-KRAS under doxycycline-inducible control. Data are presented as mean ± SD of three technical replicates. (B) Cell cycle profiles analyzed by propidium iodide staining and flow cytometry 24 h after doxycycline treatment in the same cell lines as in (A). (C) Immunoblot analysis of KRAS, phospho-ERK, and phospho-Akt in the same cell lines as in (A), 24 h after doxycycline treatment.

Since the construct relies on CRL-mediated ubiquitination, its performance in various cellular contexts needed to be evaluated. Therefore, we tested DEG-KRAS in four pancreatic cancer cell lines: PK-1, PK-45H, T3M-4, and PANC-1. All lines except PK-45H harbored monoallelic KRAS mutations (Figure S5A) and showed variable levels of KRAS protein expression by western blotting (Figure S5B).

Next, each cell line was engineered to express DEG-KRAS under doxycycline control. Upon induction, all five lines exhibited significant inhibition of cell proliferation and arrest in cell cycle progression (Figures 5A and 5B). Immunoblot analysis confirmed robust depletion of KRAS and reduced phosphorylation of ERK, indicating effective disruption of KRAS-MAPK signaling. In contrast, phosphorylation of Akt remained largely unchanged (Figure 5C), suggesting that, unlike ERK activation, the PI3K-Akt axis may be sustained by upstream signals independent of KRAS.

Because p-Akt levels were unaffected, we hypothesized that persistent Akt activity might be sustained by extracellular adhesion-mediated signals, which are known to be prominent in pancreatic cancer. To test this, DEG-KRAS-expressing PK-1 and T3M-4 cells were cultured in suspension using ultra-low-attachment conditions. Under these non-adherent conditions, cells showed markedly enhanced sensitivity to DEG-KRAS, resulting in near-complete loss of viability (Figure S5C).

Importantly, doxycycline-induced DEG-KRAS expression exerted a growth-inhibitory effect comparable to that of MRTX1133 (Figure S5D), a potent KRAS G12D inhibitor.[31]

These results indicate that DEG-KRAS is broadly effective across multiple KRAS-mutant pancreatic cancer cell lines and that its antiproliferative effects are potentiated in contexts where adhesion-mediated survival signals are disrupted.

Growth inhibition of pancreatic cancer cells by genetically manipulated MSCs

Pancreatic cancer is characterized by a dense stromal microenvironment composed of fibroblasts and collagen-rich extracellular matrix. This desmoplastic stroma limits drug penetration and contributes to therapeutic resistance.[32] MSCs are multipotent stromal cells found in various tissues, with known potential for differentiation and immune modulation. Importantly, MSCs are known to home sites of injury, including tumor tissues,[33] and secrete a large quantity of EVs, making them attractive candidates for EV-based therapeutic delivery.[7,34] These properties prompted us to explore MSCs as vehicles for delivering DEG-KRAS to tumor cells via a bystander mechanism.

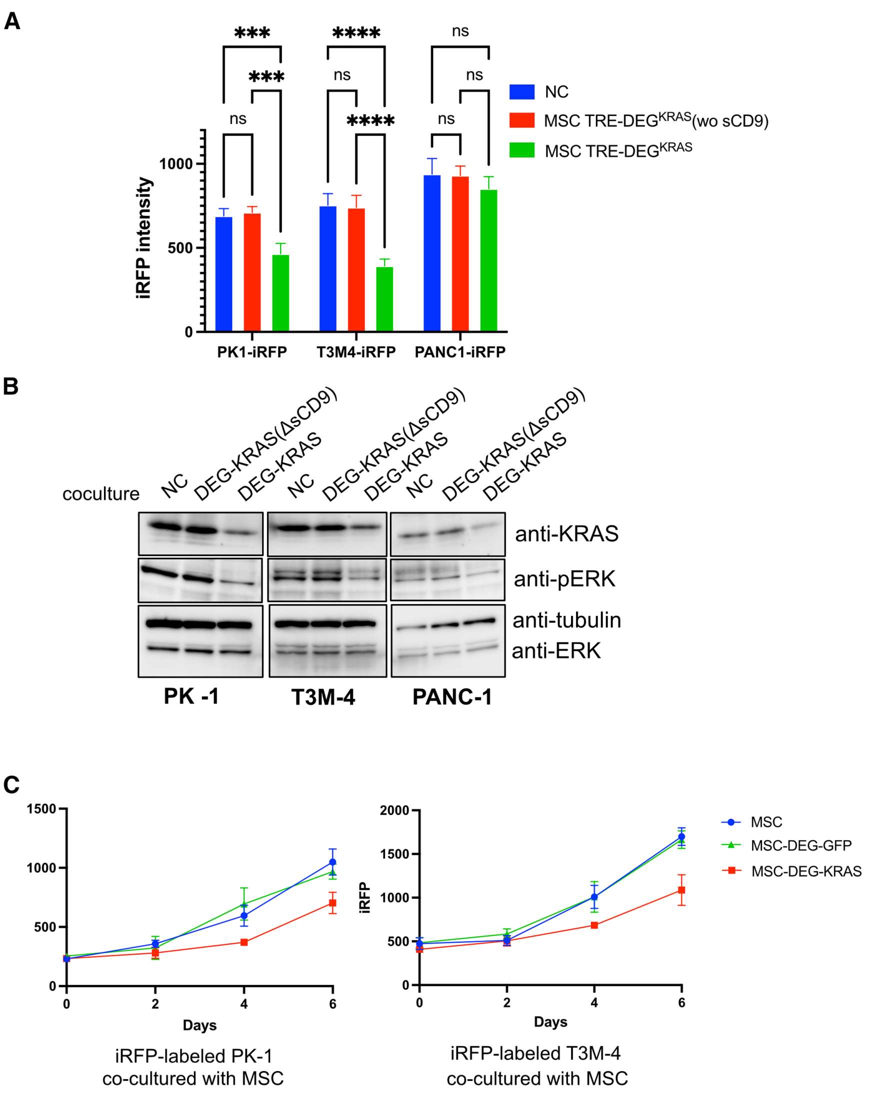

Figure 6. MSC-mediated bystander delivery of DEG-KRAS suppresses pancreatic cancer growth

(A) iRFP-labeled pancreatic cancer cells (PK-1, T3M-4, and PANC-1) co-cultured with MSCs expressing the indicated vectors. iRFP signals were measured after 120 h. (B) Immunoblot analysis of KRAS and phospho-ERK in iRFP-positive cells sorted from the co-cultures shown in (A). (C) iRFP-labeled PK-1 and T3M-4 cells co-cultured with MSCs expressing DEG-KRAS or DEG-GFP. iRFP signal intensities were measured at the indicated time points to assess proliferation. Data are presented as mean ± SD of three independent biological replicates. Statistical analysis was performed using one-way ANOVA. $^{***}p < 0.001$, $^{****}p < 0.0001$.

We first engineered telomerase-immortalized umbilical cord-derived MSCs to express DEG-KRAS in a doxycycline-inducible manner (MSC-DEG-KRAS). Western blotting confirmed successful induction of DEG-KRAS in these cells, although expression levels varied (Figure S6A). Surprisingly, neither KRAS degradation nor suppression of ERK phosphorylation was observed in this cell line (Figure S6B). Consistent with these findings, DEG-KRAS expression had minimal impact on the proliferation of MSCs themselves.

We then co-cultured MSC-DEG-KRAS with iRFP-labeled PK-1, T3M-4, and PANC-1 pancreatic cancer cells. After 5 days of co-culture, the proliferation of PK-1 and T3M-4 cancer cell lines was significantly reduced compared to controls, whereas PANC-1 cells showed no such effect. This result is consistent with the degree of KRAS dependency observed in Figure 5A, where PK-1 and T3M-4 are highly KRAS dependent, while PANC-1 cells are comparatively less dependent. (Figure 6A). Notably, this effect was not observed with a mutant lacking the exosome-targeting tag (sCD9), strongly suggesting that the growth suppression was mediated by a bystander effect. Western blot analysis of sorted iRFP-positive cells confirmed decreased levels of both KRAS and phosphorylated ERK in all cell lines (Figure 6B), indicating effective target engagement and suppression of downstream signaling in recipient cancer cells.

To rule out non-specific effects and assess temporal dynamics, we performed an additional co-culture experiment using DEG-GFP as a control. PK-1 and T3M-4 cells expressing iRFP were co-cultured with MSCs expressing either DEG-GFP or DEG-KRAS, and iRFP signal was monitored over time. A modest reduction in iRFP signal was observed only in the presence of MSC-DEG-KRAS, confirming the specificity of the bystander effect (Figure 6C). Although the antiproliferative effect was less pronounced than in monoculture conditions (Figure 5A), it remained measurable and target specific.

Collectively, these findings demonstrate that MSCs can serve as effective delivery vehicles for EV-associated bioPROTACs, enabling selective degradation of oncogenic KRAS in neighboring tumor cells. This supports the broader applicability of our system to tumor types that are difficult to target directly and highlights its potential integration into cell- or virus-based gene therapy platforms.

DISCUSSION

In this study, we developed a chimeric protein, DEG-GFP, capable of degrading a target protein and inducing bystander effects in neighboring cells. Clear bystander activity was observed in co-culture systems, whereas treatment with isolated EVs resulted in a more modest effect. Nevertheless, the degradation of the target protein was still detectable following EV treatment, suggesting that EVs may at least

partially contribute to the observed bystander effect. To further address this point, we detected HiBiT-tagged DEG-KRAS in purified EVs, confirmed its intercellular transfer to recipient cells after co-culture, and demonstrated that inhibition of EV release with GW4869 attenuated the bystander effect. These findings indicate that EV-mediated transport contributes to, though does not fully account for, the observed bystander activity. The limited potency of isolated EVs may reflect reduced uptake efficiency or degradation of cargo proteins before endosomal escape. Further investigation is warranted not only to clarify the mechanism of EV-mediated delivery but also to definitively establish the nature and extent of the bystander effect.

By replacing the GFP-binding nanobody with the RBD-CRD domains from CRAF, we redirected the system to selectively degrade activated KRAS. The choice to target the active GTP-bound form of KRAS was based on the rationale that wild-type KRAS can partially compensate for mutant-specific inhibition.[35] Although KRAS, HRAS, and NRAS share highly conserved sequences throughout the G-domain, with major differences confined to their C-terminal hypervariable regions,[36] we initially hypothesized that the CRAF-derived degrader module in DEG-KRAS might target all RAS isoforms similarly. However, our results demonstrated a preference for KRAS degradation over HRAS or NRAS. This selectivity is unlikely due to differences in degrader activity per se, but rather may reflect distinct subcellular localization patterns among RAS isoforms.[37] KRAS, particularly the KRAS4B variant, predominantly localizes to non-raft regions of the plasma membrane, whereas HRAS and NRAS are enriched in endomembrane or lipid raft compartments due to palmitoylation of their C-terminal residues. These localization differences likely influence the physical accessibility of DEG-KRAS to its target proteins, resulting in preferential degradation of membrane-localized KRAS.

The DEG-KRAS construct significantly suppressed proliferation in multiple pancreatic cancer cell lines and reduced ERK phosphorylation. Importantly, DEG-KRAS also exerted bystander effects in recipient cells via EV transfer, which may help address a major limitation of gene therapy—namely, the difficulty of delivering therapeutic genes to all tumor cells. However, for clinical application, it will be essential to develop more selective and efficient delivery methods for DEG-KRAS.

Interestingly, although KRAS degradation effectively halted cell proliferation, it did not induce substantial cell death under standard adherent 2D conditions. This cytostatic but non-cytotoxic outcome suggests that KRAS-depleted cells may activate compensatory survival pathways in the absence of oncogenic KRAS signaling. One such pathway may involve integrin-FAK signaling (Figure S5C). Given that FAK activates the PI3K-Akt pathway,[38] our observation is consistent with previous studies that have demonstrated KRAS-mutant cancer cells become more sensitive to PI3K inhibition following KRAS knockdown or inhibition.[39]

We further explored the role of KRAS in pancreatic cancer cell survival. In monoculture, most KRAS-mutant pancreatic cancer cell lines displayed strong KRAS dependency, consistent with previous reports.[40] Co-culture with MSCs expressing DEG-KRAS significantly suppressed the proliferation of pancreatic cancer cells. However, despite effective inhibition of ERK phosphorylation, the antiproliferative effect was incomplete. This suggests that while the bystander effect mediated by MSC-derived DEG-KRAS delivery is functionally active, it may not be sufficient to fully eliminate KRAS-dependent signaling in the tumor context. Interestingly, when DEG-KRAS was expressed within MSCs themselves, neither KRAS degradation nor ERK inhibition was observed. Although the precise mechanism remains unclear, one possible explanation is insufficient expression of components of the CRL complex, which mediates the degradation function of DEG-KRAS. Clarifying this point will be important for future optimization of the delivery system. These findings indicate that although the therapeutic potential of MSC-mediated delivery should be critically evaluated, the bystander approach remains a promising strategy for targeting difficult-to-transduce tumor cells. Notably, integrin signaling activates the FAK-PI3K-mTOR axis,[41] which may explain the persistent phospho-Akt levels we observed. While MSCs have been proposed as delivery vehicles due to their tumor-homing ability, our data suggest that they may also confer survival advantages to tumor cells, limiting therapeutic efficacy in the pancreatic cancer setting.

A potential limitation of our system is that DEG-KRAS is a mid-sized molecule without intrinsic cell membrane permeability. As such, it requires drug delivery systems (e.g., liposomes or adeno-associated virus vectors) or cell-based therapies to show effects. Further research is warranted to optimize these delivery strategies. Meanwhile, recent progress in the development of KRAS inhibitors, particularly those targeting previously "undruggable" mutant KRAS proteins, has led to the emergence of orally available agents with strong clinical promise.[42–44] In contrast, our approach may offer distinct advantages by targeting activated KRAS regardless of its mutation status, including wild-type KRAS, and could thus avoid compensatory mechanisms involving the wild-type protein. Nevertheless, KRAS inhibition alone may be insufficient in some settings, as cancer cells often activate parallel survival pathways. Therefore, combination strategies—such as co-targeting PI3K, FAK, or other compensatory nodes—may be required to achieve durable therapeutic responses.

In conclusion, we have established a modular degrader system that enables targeted protein degradation and exerts bystander effects on neighboring cells. While the precise mode of intercellular transfer remains to be clarified, our findings suggest a potential role for extracellular pathways such as EVs. This platform holds promise for targeting undruggable proteins such as KRAS and may be adapted to other disease-relevant targets by modifying the binding domain. When combined with appropriate cellular or viral delivery systems, this approach could serve as a foundation for next-generation, non-cell-autonomous protein degradation therapies.

MATERIAL AND METHODS

Plasmid construction

All plasmids were constructed using InFusion (Takara) or LR Clonase II (Thermo Fisher) according to the manufacturer's instructions. CSIV-TRE-RfA-CMV-KT (RDB12876, Riken) was used as a destination vector in the LR Clonase reaction (Thermo Fisher) for lentiviral production, and PB-TAG-ERP2 and PB TAC-ERP2 (gifts from Knut Woltjen [Addgene plasmid # 80479 and # 80478]) were used for piggyBac vector production.[45]

Cells

293T cells (purchased from Takara Bio Inc., Shiga, Japan) and HeLa cells (obtained from the Japanese Collection of Research Bioresources Cell Bank, JCRB) were cultured in DMEM with low glucose (Thermo Fisher) supplemented with 10% fetal bovine serum (FBS) and 100 U/mL penicillin/streptomycin. Cells were cultured in advanced DMEM (Thermo Fisher) supplemented with 3% EV-depleted FBS to produce EVs for downstream assays. MSCs were purchased from ATCC (ASC 52-telo) and maintained in Cellartis MSC Xeno-Free Culture Medium (Takara) containing 100 μg/mL G-418 (InvivoGen). Pancreatic cancer cell lines (PK-1, PK-45H, T3M-4, and PANC-1) were purchased from the Japanese Collection of Research Bioresources Cell Bank and cultured in RMPI1640 supplemented with 10% FBS and 100 U/mL penicillin/strep. Cells were seeded in EZ-BindShut 96-well plates (Iwaki) for suspension culture.

Transfection and generation of stable cell lines

Stable cell lines derived from 293T, HeLa, and pancreatic cancer cells were generated using the piggyBac vector system. Cells were seeded on the day before transfection. At 50%–70% confluency, the cells were transfected with the indicated plasmids using Avalanche transfection reagent (EZ Bioscience) according to the manufacturer's instructions. A DNA mixture containing a piggyBac vector and transposase (5:2 molar ratio) was used, followed by selection with 1 μg/mL puromycin or 10 μg/mL blasticidin 24 h after transfection to generate stable cell lines. For MSCs, lentiviral infection was selected. Lentiviral production was performed as described previously.[46] Generated cell lines were maintained in a medium containing 1 μg/mL puromycin or 10 μg/mL blasticidin.

EV depletion from fetal calf serum

FBS (Gibco) was centrifuged at 100,000 × *g* for 12 h (TLA-50 rotor in Optima-MAX-XP, Beckmann Coulter). Subsequently, the supernatant was processed using a 0.45-μm syringe filter (polyether sulfone, Sartorius).

EV isolation, nanotracking analysis, and quantification

293T cells were cultured in advanced DMEM supplemented with 3% EV-depleted FBS for 48 h. Culture supernatants were harvested and centrifuged at 1,200 × g for 3 min to remove cells and large debris, followed by filtration through a 0.45-μm syringe filter (polyethersulfone, Sartorius). The filtrate was concentrated using a 100-kDa molecular weight cutoff ultrafiltration unit (Vivaspin 20, Sartorius), and buffer was exchanged to PBS. The concentrated supernatant was layered onto a 30% sucrose cushion (w/v in PBS) and ultracentrifuged at 100,000 × *g* for 60 min at 4°C using an MLS-50 rotor (Optima MAX-XP, Beckman Coulter). The EV-containing sucrose interface was carefully collected and subjected to buffer exchange with PBS using a 100-kDa molecular weight cutoff ultrafiltration device (Vivaspin 500, Sartorius) prior to downstream analysis.

Measurement of HiBiT signals

Cells were cultured in a white 96-well flat bottom plate (Corning) containing 100 μL medium. The medium was aspirated, cells were washed with 100 μL PBS twice, and 100 μL HiBiT lytic detection reagent (Promega) was directly added to the plate to measure cellular HiBiT signals. After 15 min of incubation at room temperature (RT), luminescent signals were obtained using a plate reader (Enspire, PerkinElmer). The culture medium was transferred to microtubes and centrifuged at 20,000 × *g* for 10 min to measure HiBiT signals in EVs. CellTiter Glo (Promega) was measured in separate wells according to the manufacturer's instructions to adjust the HiBiT signal by cell number, and the HiBiT/CellTiter ratio was analyzed. Remark: units of measure that accompany numerical values must be appropriately abbreviated. For bystander effect assays to demonstrate degrader proteins, (e.g., Figure 4E), HiBiT was fused to the target protein, allowing luminescence signals to reflect the number of target molecules. HiBiT and CellTiter signals were measured from the entire co-culture population. The proportion of mCherry-negative recipient cells was determined by flow cytometry and used to estimate the recipient-specific CellTiter value, and the HiBiT signal was normalized.

Quantification of iRFP-positive cells

iRFP-positive pancreatic cancer cells were cultured in 6-well plates with DEG^{KRAS} provider cells. Cells were collected by trypsinization and washed with PBS three times. The iRFP signals of the cell pellets were quantified (Pearl Impulse, LI-COR).

Cell cycle analysis

Cells were washed and resuspended at 1 × 10E6 cells/mL in a 15-mL conical tube, and 2.5× volume of 100% ethanol was added dropwise while gently vortexing and incubated for 1 h. Cells were centrifuged at 700 × *g* for 10 min and washed twice with PBS. Cell pellets were resuspended with 1 mL PBS containing 50 g/mL propidium iodide (PI). Cells were analyzed by flow cytometry after incubation at 4°C for 4 h.

Immunoblotting and immunoprecipitation

Cells were trypsinized and resuspended in PBS. The same number of cells was transferred to the microtubes. Cell pellets were lysed in radioimmunoprecipitation assay buffer (50 mmol/L Tris-HCl buffer (pH 7.6), 150 mM NaCl, 1% Nonidet P40 substitute, 0.5% sodium deoxycholate, and 0.1% SDS) supplemented with a protease inhibitor cocktail on ice for 10 min. The lysates were briefly sonicated for 10 min (Bioruptor, BM Bio) and centrifuged at 14,000 × *g* for

10 min. The supernatant was analyzed by immunoblotting, as previously described.[46] Table S2 lists the antibodies used.

Immunocytochemistry

Cells were grown on a cover glass, washed twice with PBS, and fixed in PBS containing 4% paraformaldehyde for 10 min. Cells were permeabilized with 0.5% Triton X-100/PBS for 5 min. After blocking with 2% BSA/PBS at RT for 30 min, the samples were stained with antibodies diluted in 2% BSA/PBS for 1 h at RT, followed by washing with PBS three times and immunostaining with secondary antibodies in 2% BSA/PBS for 1 h at RT (Alexa Fluor 488-conjugated anti-mouse IgG and Alexa Fluor 555-conjugated anti-rabbit IgG [Molecular Probes]). Table S2 shows the antibodies used.

Flow cytometry and cell sorting

Flow cytometry was performed using a CytoFLEX flow cytometer (Beckman Coulter). EGFP fluorescence was detected using a 488-nm laser with a 525/40-nm filter, and iRFP was detected using a 638-nm laser with a 660/20-nm filter. Cell cycle analysis was performed using PI fluorescence in linear mode. Data were analyzed using CytExpert software. For MSC co-culture experiments, iRFP-positive cells were sorted to isolate pancreatic cancer cells (SONY SH-800).

Fluorescence microscopy

Fluorescence imaging was conducted using the EVOS FL Auto 2 imaging system (Thermo Fisher Scientific) equipped with a 60× Plan Fluor oil-immersion objective. GFP and mCherry channels were used to visualize EGFP-KRAS and DEG constructs. All images were captured with constant exposure settings and processed using the EVOS software.

Protein quantification

Protein concentration of cell lysates was determined using the BCA Protein Assay Kit (Thermo Fisher Scientific) before SDS-PAGE and immunoblotting.

Statistical analysis

Experiments were independently repeated at least three times unless otherwise stated. Data are presented as mean ± SEM. Statistical significance was determined using unpaired two-tailed Student's t test or one-way ANOVA followed by Tukey's multiple comparisons test, as appropriate. Statistical analysis and graphing were performed using GraphPad Prism 9 software. A p value <0.05 was considered statistically significant. p values are denoted as follows: $^{*}p < 0.05$, $^{**}p < 0.01$, $^{***}p < 0.001$, $^{****}p < 0.0001$.

DATA AND CODE AVAILABILITY

The datasets generated for this study are available on request to the corresponding author.

ACKNOWLEDGMENTS

The authors thank the research division of Medical Research Institute Kitano Hospital, especially the Director Mark Makoto Taketo, for their continuous support. This work was supported by the Japan Society for the Promotion of Science (JSPS) KAKENHI grant number 22K15570.

AUTHOR CONTRIBUTIONS

S.I. conceived and designed the study, performed experiments, analyzed the data, and wrote the manuscript. A.T.K. and T.N. supervised the study and provided critical feedback on experimental strategy and manuscript revision. All authors read and approved the final version of the manuscript.

DECLARATION OF INTERESTS

The authors declare no competing interests.

DECLARATION OF GENERATIVE AI AND AI-ASSISTED TECHNOLOGIES IN THE WRITING PROCESS

During the preparation of this work the authors used ChatGPT(GPT-5, OpenAI) in order to assist in language editing of this manuscript. After using this tool, the authors reviewed and edited the content as needed and take full responsibility for the content of the published article.

SUPPLEMENTAL INFORMATION

Supplemental information can be found online at https://doi.org/10.1016/j.omton.2025.201077.

REFERENCES

1. Cohen, P., Cross, D., and Jänne, P.A. (2021). Kinase drug discovery 20 years after imatinib: progress and future directions. Nat. Rev. Drug Discov. *20*, 551–569. https://doi.org/10.1038/s41573-021-00195-4.
2. DiNardo, C.D., Jonas, B.A., Pullarkat, V., Thirman, M.J., Garcia, J.S., Wei, A.H., Konopleva, M., Döhner, H., Letai, A., Fenaux, P., et al. (2020). Azacitidine and Venetoclax in Previously Untreated Acute Myeloid Leukemia. N. Engl. J. Med. *383*, 617–629. https://doi.org/10.1056/nejmoa2012971.
3. Shorstova, T., Foulkes, W.D., and Witcher, M. (2021). Achieving clinical success with BET inhibitors as anti-cancer agents. Br. J. Cancer *124*, 1478–1490. https://doi.org/10.1038/s41416-021-01321-0.
4. Békés, M., Langley, D.R., and Crews, C.M. (2022). PROTAC targeted protein degraders: the past is prologue. Nat. Rev. Drug Discov. *21*, 181–200. https://doi.org/10.1038/s41573-021-00371-6.
5. Lim, S., Khoo, R., Peh, K.M., Teo, J., Chang, S.C., Ng, S., Beilhartz, G.L., Melnyk, R.A., Johannes, C.W., Brown, C.J., et al. (2020). bioPROTACs as versatile modulators of intracellular therapeutic targets including proliferating cell nuclear antigen (PCNA). Proc. Natl. Acad. Sci. *117*, 5791–5800. https://doi.org/10.1073/pnas.1920251117.
6. Kalluri, R., and LeBleu, V.S. (2020). The biology, function, and biomedical applications of exosomes. Science *367*, eaau6977. https://doi.org/10.1126/science.aau6977.
7. Tang, Y., Zhou, Y., and Li, H.-J. (2021). Advances in mesenchymal stem cell exosomes: a review. Stem Cell Res. Ther. *12*, 71. https://doi.org/10.1186/s13287-021-02138-7.
8. Xu, S., Menu, E., De Becker, A., Van Camp, B., Vanderkerken, K., and Van Riet, I. (2012). Bone Marrow-Derived Mesenchymal Stromal Cells are Attracted by Multiple Myeloma Cell-Produced Chemokine CCL25 and Favor Myeloma Cell Growth in Vitro and In Vivo. STEM CELLS *30*, 266–279. https://doi.org/10.1002/stem.787.
9. Phinney, D.G., and Pittenger, M.F. (2017). Concise Review: MSC-Derived Exosomes for Cell-Free Therapy. STEM CELLS *35*, 851–858. https://doi.org/10.1002/stem.2575.
10. Gurunathan, S., Kang, M.-H., Jeyaraj, M., Qasim, M., and Kim, J.-H. (2019). Review of the Isolation, Characterization, Biological Function, and Multifarious Therapeutic Approaches of Exosomes. Cells *8*, 307. https://doi.org/10.3390/cells8040307.
11. Ruivo, C.F., Adem, B., Silva, M., and Melo, S.A. (2017). The Biology of Cancer Exosomes: Insights and New Perspectives. Cancer Res. *77*, 6480–6488. https://doi.org/10.1158/0008-5472.can-17-0994.
12. Shen, B., Wu, N., Yang, J.-M., and Gould, S.J. (2011). Protein Targeting to Exosomes/Microvesicles by Plasma Membrane Anchors. J. Biol. Chem. *286*, 14383–14395. https://doi.org/10.1074/jbc.m110.208660.

13. Bery, N., Keller, L., Soulié, M., Gence, R., Iscache, A.-L., Cherier, J., Cabantous, S., Sordet, O., Lajoie-Mazenc, I., Pedelacq, J.-D., et al. (2019). A Targeted Protein Degradation Cell-Based Screening for Nanobodies Selective toward the Cellular RHOB GTP-Bound Conformation. Cell Chem. Biol. *26*, 1544–1558.e6. https://doi.org/10.1016/j.chembiol.2019.08.009.

14. Fulcher, L.J., Macartney, T., Bozatzi, P., Hornberger, A., Rojas-Fernandez, A., and Sapkota, G.P. (2016). An affinity-directed protein missile system for targeted proteolysis. Open Biol. *6*, 160255. https://doi.org/10.1098/rsob.160255.

15. Muyldermans, S. (2013). Nanobodies: Natural Single-Domain Antibodies. Annu. Rev. Biochem. *82*, 775–797. https://doi.org/10.1146/annurev-biochem-063011-092449.

16. Kubala, M.H., Kovtun, O., Alexandrov, K., and Collins, B.M. (2010). Structural and thermodynamic analysis of the GFP:GFP-nanobody complex. Protein Sci. *19*, 2389–2401. https://doi.org/10.1002/pro.519.

17. de Bie, P., and Ciechanover, A. (2011). Ubiquitination of E3 ligases: self-regulation of the ubiquitin system via proteolytic and non-proteolytic mechanisms. Cell Death Differ. *18*, 1393–1402. https://doi.org/10.1038/cdd.2011.16.

18. Reyes, R., Cardeñes, B., Machado-Pineda, Y., and Cabañas, C. (2018). Tetraspanin CD9: A Key Regulator of Cell Adhesion in the Immune System. Front. Immunol. *9*, 863. https://doi.org/10.3389/fimmu.2018.00863.

19. Andreu, Z., and Yáñez-Mó, M. (2014). Tetraspanins in Extracellular Vesicle Formation and Function. Front. Immunol. *5*, 442. https://doi.org/10.3389/fimmu.2014.00442.

20. Inano, S., and Kitano, T. (2024). A modified CD9 tag for efficient protein delivery via extracellular vesicles. PLoS One *19*, e0310083. https://doi.org/10.1371/journal.pone.0310083.

21. Varkouhi, A.K., Scholte, M., Storm, G., and Haisma, H.J. (2011). Endosomal escape pathways for delivery of biologicals. J. Control. Release *151*, 220–228. https://doi.org/10.1016/j.jconrel.2010.11.004.

22. Waters, A.M., and Der, C.J. (2018). KRAS: The Critical Driver and Therapeutic Target for Pancreatic Cancer. Csh Perspect Med *8*, a031435. https://doi.org/10.1101/cshperspect.a031435.

23. Collins, M.A., Bednar, F., Zhang, Y., Brisset, J.-C., Galbán, S., Galbán, C.J., Rakshit, S., Flannagan, K.S., Adsay, N.V., and Pasca di Magliano, M. (2012). Oncogenic Kras is required for both the initiation and maintenance of pancreatic cancer in mice. J. Clin. Investig. *122*, 639–653. https://doi.org/10.1172/jci59227.

24. Biankin, A.V., Waddell, N., Kassahn, K.S., Gingras, M.-C., Muthuswamy, L.B., Johns, A.L., Miller, D.K., Wilson, P.J., Patch, A.-M., Wu, J., et al. (2012). Pancreatic cancer genomes reveal aberrations in axon guidance pathway genes. Nature *491*, 399–405. https://doi.org/10.1038/nature11547.

25. Teo, S.L.Y., Rennick, J.J., Yuen, D., Al-Wassiti, H., Johnston, A.P.R., and Pouton, C.W. (2021). Unravelling cytosolic delivery of cell penetrating peptides with a quantitative endosomal escape assay. Nat. Commun. *12*, 3721. https://doi.org/10.1038/s41467-021-23997-x.

26. Boilève, A., Smolenschi, C., Lambert, A., Boige, V., Delaye, M., Camilleri, G.M., Tarabay, A., Valéry, M., Fuerea, A., Pudlarz, T., et al. (2024). KRAS, a New Target for Precision Medicine in Colorectal Cancer? Cancers *16*, 3455. https://doi.org/10.3390/cancers16203455.

27. Bryant, K.L., Mancias, J.D., Kimmelman, A.C., and Der, C.J. (2014). KRAS: feeding pancreatic cancer proliferation. Trends Biochem. Sci. *39*, 91–100. https://doi.org/10.1016/j.tibs.2013.12.004.

28. Hibino, K., Shibata, T., Yanagida, T., and Sako, Y. (2011). Activation Kinetics of RAF Protein in the Ternary Complex of RAF, RAS-GTP, and Kinase on the Plasma Membrane of Living Cells SINGLE-MOLECULE IMAGING ANALYSIS. J. Biol. Chem. *286*, 36460–36468. https://doi.org/10.1074/jbc.m111.262675.

29. Holderfield, M., Deuker, M.M., McCormick, F., and McMahon, M. (2014). Targeting RAF kinases for cancer therapy: BRAF-mutated melanoma and beyond. Nat. Rev. Cancer *14*, 455–467. https://doi.org/10.1038/nrc3760.

30. Yin, G., Kistler, S., George, S.D., Kuhlmann, N., Garvey, L., Huynh, M., Bagni, R.K., Lammers, M., Der, C.J., and Campbell, S.L. (2017). A KRAS GTPase K104Q Mutant Retains Downstream Signaling by Offsetting Defects in Regulation. J. Biol. Chem. *292*, 4446–4456. https://doi.org/10.1074/jbc.m116.762435.

31. Kemp, S.B., Cheng, N., Markosyan, N., Sor, R., Kim, I.-K., Hallin, J., Shoush, J., Quinones, L., Brown, N.V., Bassett, J.B., et al. (2023). Efficacy of a Small-Molecule Inhibitor of KrasG12D in Immunocompetent Models of Pancreatic Cancer. Cancer Discov. *13*, 298–311. https://doi.org/10.1158/2159-8290.cd-22-1066.

32. Hosein, A.N., Brekken, R.A., and Maitra, A. (2020). Pancreatic cancer stroma: an update on therapeutic targeting strategies. Nat. Rev. Gastroenterol. Hepatol. *17*, 487–505. https://doi.org/10.1038/s41575-020-0300-1.

33. El Agha, E., Kramann, R., Schneider, R.K., Li, X., Seeger, W., Humphreys, B.D., and Bellusci, S. (2017). Mesenchymal Stem Cells in Fibrotic Disease. Cell Stem Cell *21*, 166–177. https://doi.org/10.1016/j.stem.2017.07.011.

34. Muntión, S., Ramos, T.L., Diez-Campelo, M., Rosón, B., Sánchez-Abarca, L.I., Misiewicz-Krzeminska, I., Preciado, S., Sarasquete, M.-E., de Las Rivas, J., González, M., et al. (2016). Microvesicles from Mesenchymal Stromal Cells Are Involved in HPC-Microenvironment Crosstalk in Myelodysplastic Patients. PLoS One *11*, e0146722. https://doi.org/10.1371/journal.pone.0146722.

35. Xue, J.Y., Zhao, Y., Aronowitz, J., Mai, T.T., Vides, A., Qeriqi, B., Kim, D., Li, C., de Stanchina, E., Mazutis, L., et al. (2020). Rapid non-uniform adaptation to conformation-specific KRAS(G12C) inhibition. Nature *577*, 421–425. https://doi.org/10.1038/s41586-019-1884-x.

36. Castellano, E., and Santos, E. (2011). Functional Specificity of Ras Isoforms: So Similar but So Different. Genes Cancer *2*, 216–231. https://doi.org/10.1177/1947601911408081.

37. Prior, I.A., and Hancock, J.F. (2012). Ras trafficking, localization and compartmentalized signalling. Semin. Cell Dev. Biol. *23*, 145–153. https://doi.org/10.1016/j.semcdb.2011.09.002.

38. Mitra, S.K., Hanson, D.A., and Schlaepfer, D.D. (2005). Focal adhesion kinase: in command and control of cell motility. Nat. Rev. Mol. Cell Biol. *6*, 56–68. https://doi.org/10.1038/nrm1549.

39. Muzumdar, M.D., Chen, P.-Y., Dorans, K.J., Chung, K.M., Bhutkar, A., Hong, E., Noll, E.M., Sprick, M.R., Trumpp, A., and Jacks, T. (2017). Survival of pancreatic cancer cells lacking KRAS function. Nat. Commun. *8*, 1090. https://doi.org/10.1038/s41467-017-00942-5.

40. Collins, M.A., Brisset, J.-C., Zhang, Y., Bednar, F., Pierre, J., Heist, K.A., Galbán, C.J., Galbán, S., and di Magliano, M.P. (2012). Metastatic Pancreatic Cancer Is Dependent on Oncogenic Kras in Mice. PLoS One *7*, e49707. https://doi.org/10.1371/journal.pone.0049707.

41. Desgrosellier, J.S., and Cheresh, D.A. (2010). Integrins in cancer: biological implications and therapeutic opportunities. Nat. Rev. Cancer *10*, 9–22. https://doi.org/10.1038/nrc2748.

42. Kim, T.W., Price, T., Grasselli, J., Strickler, J.H., Masuishi, T., Kwok, G.W., Yalcin, S., Obiozor, C.C., Chan, E., Gokani, P., and Sunakawa, Y. (2025). A phase 3 study of first-line sotorasib, panitumumab, and FOLFIRI versus FOLFIRI with or without bevacizumab-awwb for patients with KRAS G12C–mutated metastatic colorectal cancer (CodeBreaK 301). J. Clin. Oncol. *43*, TPS326. https://doi.org/10.1200/jco.2025.43.4_suppl.tps326.

43. Hallin, J., Bowcut, V., Calinisan, A., Briere, D.M., Hargis, L., Engstrom, L.D., Laguer, J., Medwid, J., Vanderpool, D., Lifset, E., et al. (2022). Anti-tumor efficacy of a potent and selective non-covalent KRASG12D inhibitor. Nat. Med. *28*, 2171–2182. https://doi.org/10.1038/s41591-022-02007-7.

44. Hallin, J., Engstrom, L.D., Hargis, L., Calinisan, A., Aranda, R., Briere, D.M., Sudhakar, N., Bowcut, V., Baer, B.R., Ballard, J.A., et al. (2020). The KRASG12C Inhibitor MRTX849 Provides Insight toward Therapeutic Susceptibility of KRAS-Mutant Cancers in Mouse Models and Patients. Cancer Discov. *10*, 54–71. https://doi.org/10.1158/2159-8290.cd-19-1167.

45. Kim, S.-I., Oceguera-Yanez, F., Sakurai, C., Nakagawa, M., Yamanaka, S., and Woltjen, K. (2016). Induced Pluripotent Stem (iPS) Cells, Methods and Protocols. Methods Mol. Biol. *1357*, 111–131. https://doi.org/10.1007/7651_2015_251.

46. Inano, S., Sato, K., Katsuki, Y., Kobayashi, W., Tanaka, H., Nakajima, K., Nakada, S., Miyoshi, H., Knies, K., Takaori-Kondo, A., et al. (2017). RFWD3-Mediated Ubiquitination Promotes Timely Removal of Both RPA and RAD51 from DNA Damage Sites to Facilitate Homologous Recombination. Mol. Cell *66*, 622–634.e8. https://doi.org/10.1016/j.molcel.2017.04.022.

OMTON, Volume *33*

Supplemental information

Targeted degradation of KRAS and induction of bystander effects by a modular bioPROTAC

Shojiro Inano, Akifumi Takaori-Kondo, and Takako Nakajima

Supplemental Information

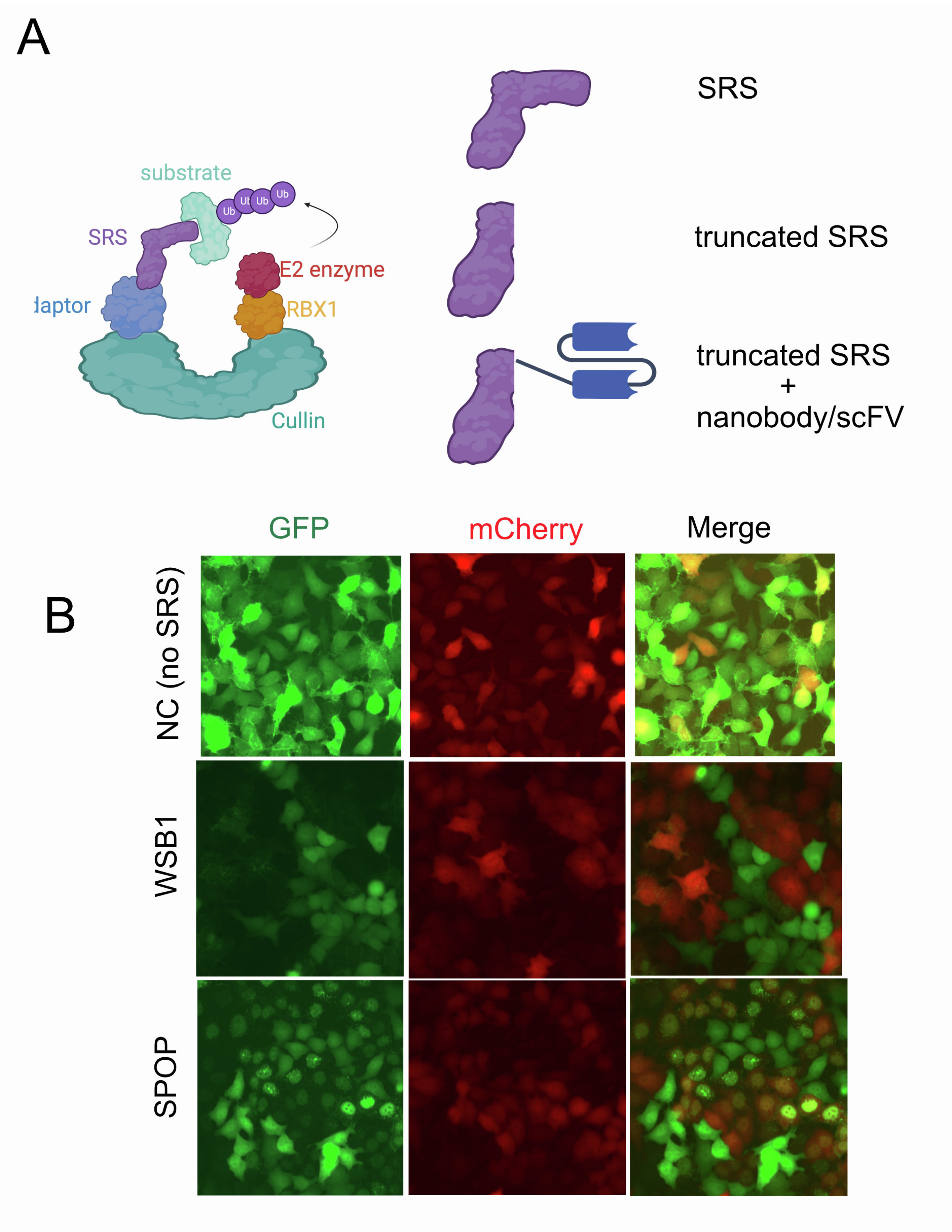

Figure S1. Screening of substrate recognition subunits (SRS) and visualization of EGFP degradation. (A) Schematic of the cullin-RING ligase (CRL) complex and representative substrate recognition subunits (SRS) selected for this study. The SRS consists of an adaptor-binding domain and a target-binding domain. By truncating the native target-binding domain and replacing it with an scFv or VHH, the system can be redirected to degrade arbitrary targets. In this study, a GFP nanobody was used as the target-binding module. (B) Fluorescence microscopy showing EGFP signal reduction in 293T cells expressing HiBiT–EGFP–KRAS and GFP degraders with various SRSs (labeled with mCherry).

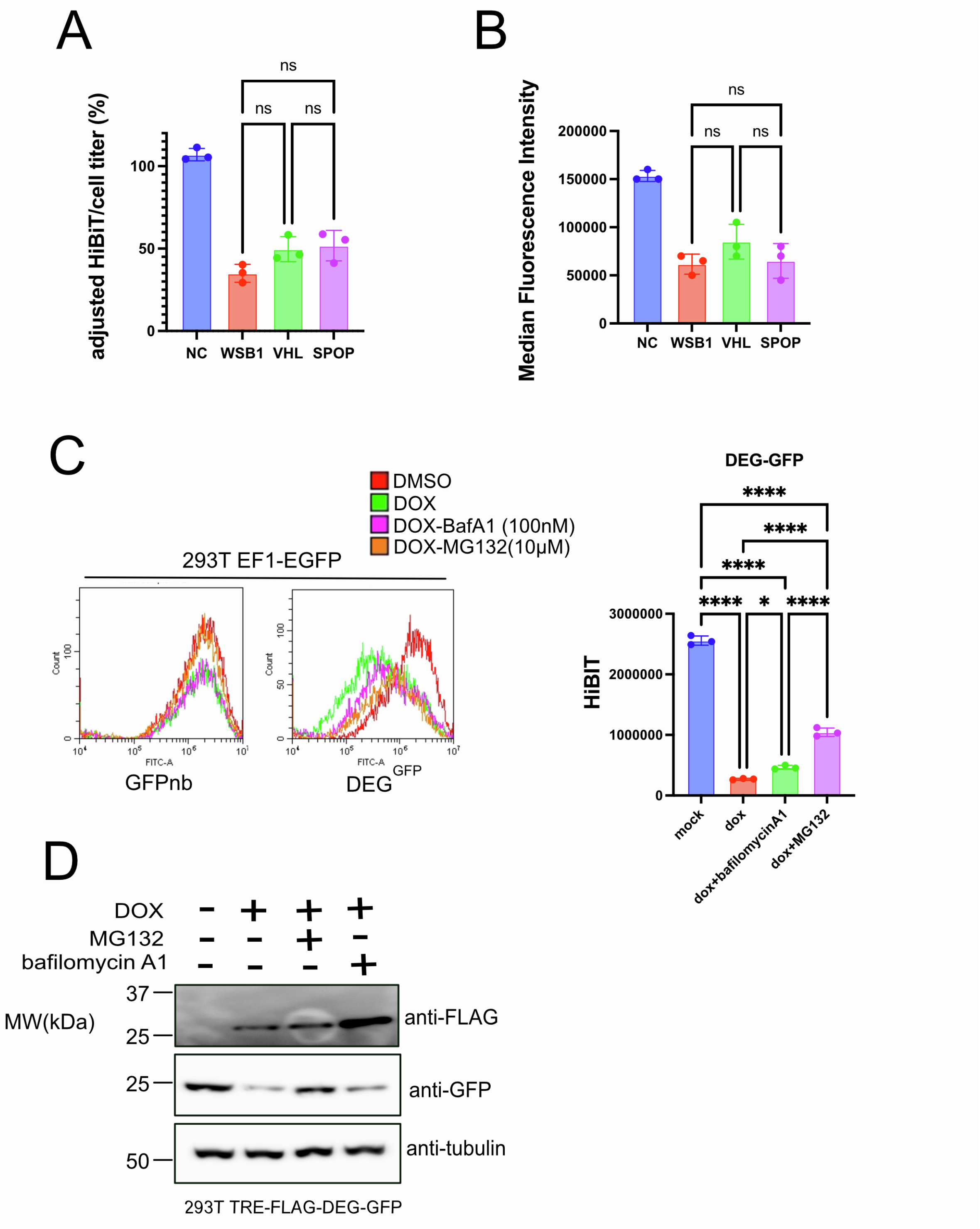
A
adjusted HiBiT/cell titer (%)
100
50
0
NC
WSB1
VHL
SPOP
ns
B
Median Fluorescence Intensity
200000
150000
100000
50000
0
NC
WSB1
VHL
SPOP
ns
C
DMSO
DOX
DOX-BafA1 (100nM)
DOX-MG132(10μM)
293T EF1-EGFP
Count
FITC-A
GFPnb
DEG GFP
DEG-GFP
****
*
HiBIT
3000000
2000000
1000000
0
mock
dox
dox+bafilomycinA1
dox+MG132
D
DOX
MG132
bafilomycin A1
MW(kDa)
37
25
50
anti-FLAG
anti-GFP
anti-tubulin
293T TRE-FLAG-DEG-GFP

Figure S2. Functional characterization of SRS constructs and degradation pathways.

(A, B) Flow cytometry (A) and HiBiT assays (B) showing HiBiT-EGFP degradation in 293T cells expressing GFP nanobody–sCD9-SRS proteins (WSB1, VHL, SPOP). No statistically significant differences in degradation efficiency were observed between the constructs, although WSB1 showed a trend toward higher efficiency. (C) Both bafilomycin A1 and MG132 partially inhibited the degradation of DEG-GFP (GFPnanobody-sCD9-WSB1), with MG132 showing a significantly stronger inhibitory effect. Experiments were performed in triplicate, and representative flow cytometry plots along with the median ± SD are shown. (D) Cells in (C) are collected analyzed by immunoblotting. Statistical analysis was performed using one-way ANOVA to compare differences between groups. Data are presented as mean ± SD of three independent biological replicates. Statistical analysis was performed using one-way ANOVA. Data are presented as mean ± SD. $p < 0.05$ (*), $p < 0.01$ (**), $p < 0.001$ (***), $p < 0.0001$ (****).

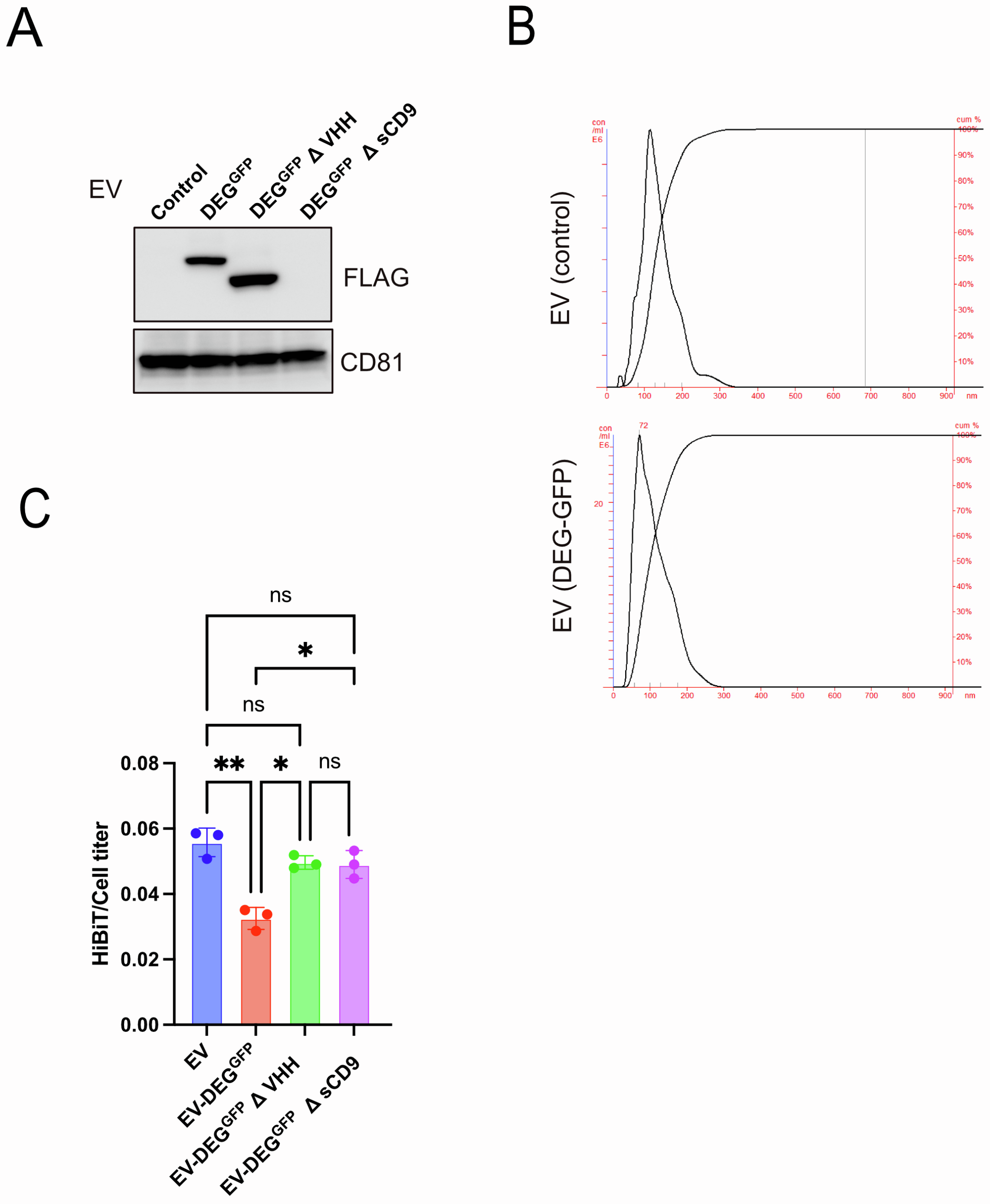
A
B
C
EV
Control
DEG^GFP
DEG^GFP Δ VHH
DEG^GFP Δ sCD9
FLAG
CD81
EV (control)
EV (DEG-GFP)
ns
*
ns
**
*
ns
HiBiT/Cell titer
0.08
0.06
0.04
0.02
0.00
EV
EV-DEG^GFP
EV-DEG^GFP Δ VHH
EV-DEG^GFP Δ sCD9

Figure S3. EV loading of DEG-GFP constructs and functional activity.

(A) Western blot analysis of EVs purified from 293T cells transiently expressing full-length DEG-GFP or deletion mutants lacking the GFP nanobody (ΔVHH), sCD9 (ΔsCD9). EV markers (CD81) and degrader constructs were detected. The ΔsCD9 mutant showed minimal EV incorporation, whereas ΔVHH constructs were successfully loaded.

(B) Nanoparticle Tracking Analysis (NTA) of extracellular vesicles (EVs) isolated from culture supernatants of 293T cells transiently transfected with DEG-GFP or a negative-control vector (NC), as described in Methods. NTA, which estimates particle concentration and size distribution by tracking Brownian motion, showed two overlaid profiles corresponding to the NC and DEG-GFP samples, respectively, with nearly identical size distributions and comparable particle concentrations. These data indicate that sCD9-mediated cargo loading does not substantially alter EV production. Shown is one representative result from two independent experiments.

(C) Recipient 293T cells expressing HiBiT–EGFP–KRAS were treated with the EVs prepared in (A). 5μg of EVs were added per 1 x 10^5 cells. Only EVs containing full-length DEG-GFP induced a significant reduction in HiBiT signal. EVs derived from ΔVHH constructs failed to induce degradation despite successful EV incorporation, while ΔsCD9 EVs lacked effect due to impaired loading. Data are presented as mean ± SD of three independent biological replicates. Statistical analysis was performed using one-way ANOVA. $p < 0.05$ (*), $p < 0.01$ (**), $p < 0.001$ (***), $p < 0.0001$ (****).

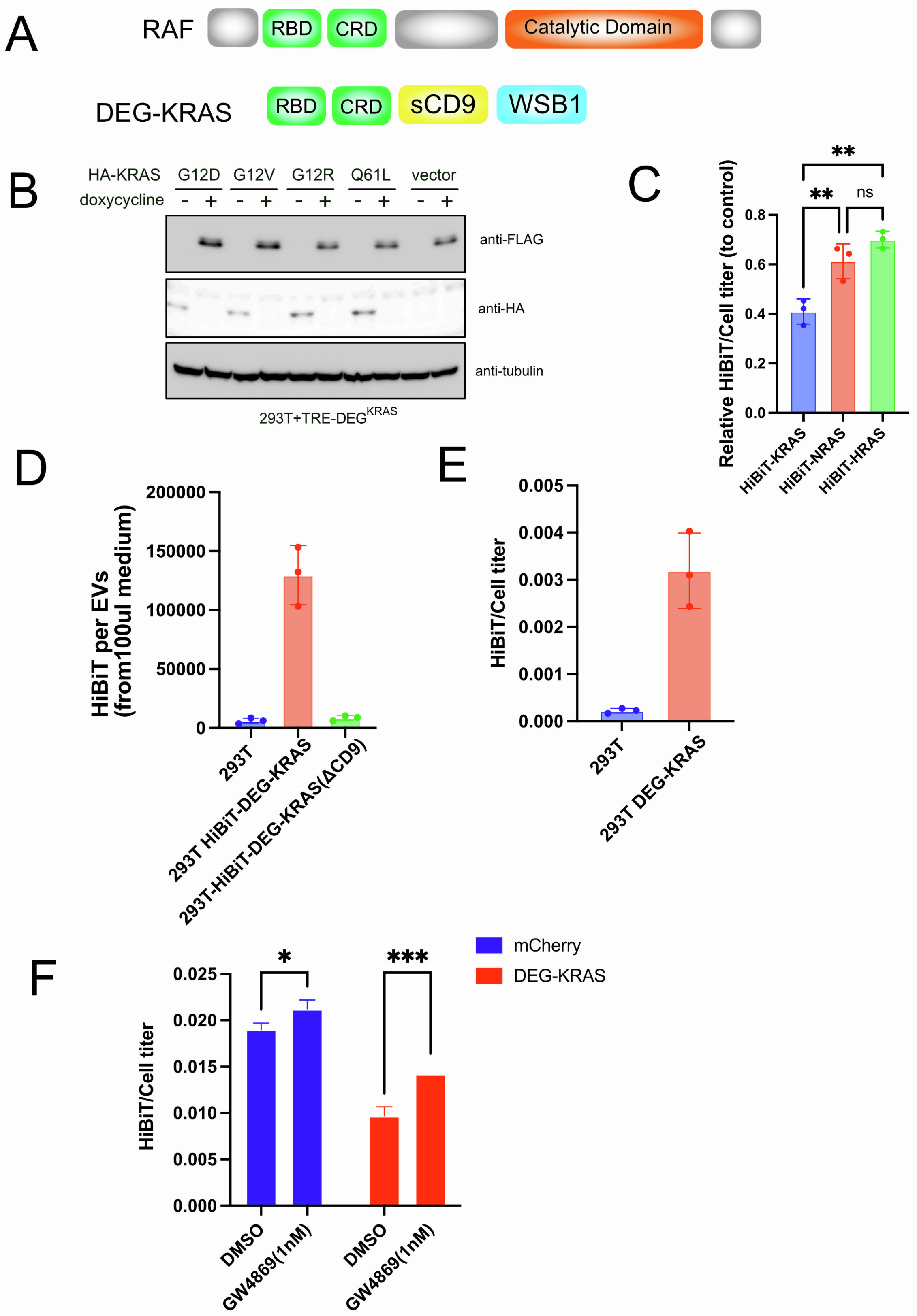
A
RAF
RBD
CRD
Catalytic Domain
DEG-KRAS
RBD
CRD
sCD9
WSB1
B
HA-KRAS
G12D
G12V
G12R
Q61L
vector
doxycycline
anti-FLAG
anti-HA
anti-tubulin
293T+TRE-DEG^KRAS
C
Relative HiBiT/Cell titer (to control)
HiBiT-KRAS
HiBiT-NRAS
HiBiT-HRAS
ns
D
HiBiT per EVs (from100ul medium)
293T
293T HiBiT-DEG-KRAS
293T-HiBiT-DEG-KRAS(ΔCD9)
E
HiBiT/Cell titer
293T
293T DEG-KRAS
F
HiBiT/Cell titer
mCherry
DEG-KRAS
DMSO
GW4869(1nM)
DMSO
GW4869(1nM)

Figure S4. EV-mediated transfer of DEG-KRAS.

(A) Domain organization of CRAF, including the Ras-binding domain (RBD) and cysteine-rich domain (CRD), used to construct DEG-KRAS.

(B) Immunoblot analysis showing KRAS degradation by DEG-KRAS across multiple KRAS mutant variants.

(C) 293T cells stably expressing HiBiT-tagged KRAS, NRAS, or HRAS were treated with doxycycline (1 μg/mL) for 24 hours to induce expression of DEG-KRAS. HiBiT luminescence was measured and normalized to cell viability (CellTiter-Glo). Bar graphs show the relative HiBiT/Cell Titer values compared to no doxycycline control.

(D) 293T–DEG-KRAS donor cells were co-cultured with iRFP-labeled recipient 293T cells in the presence or absence of the EV release inhibitor GW4869. The bystander effect, measured as reduction of HiBiT-tagged KRAS in recipient cells, was attenuated upon GW4869 treatment.

(E) 293T cells stably expressing HiBiT-tagged DEG-KRAS were cultured, and extracellular vesicles (EVs) were purified from conditioned medium. HiBiT activity was measured in the EV fraction, demonstrating secretion of DEG-KRAS via EVs.

(F) 293T donor cells stably expressing HiBiT-tagged DEG-KRAS were co-cultured with iRFP-labeled 293T recipient cells. After co-culture, recipient cells were isolated by flow cytometric sorting of the iRFP-positive population, and HiBiT activity was measured and normalized to cell titer. A small but detectable HiBiT signal was observed in recipient cells, confirming intercellular transfer of DEG-KRAS.

Data are presented as mean ± SD of three independent biological replicates. Statistical analysis was performed using one-way ANOVA. $p < 0.05$ (*), $p < 0.01$ (**), $p < 0.001$ (***), $p < 0.0001$ (****).

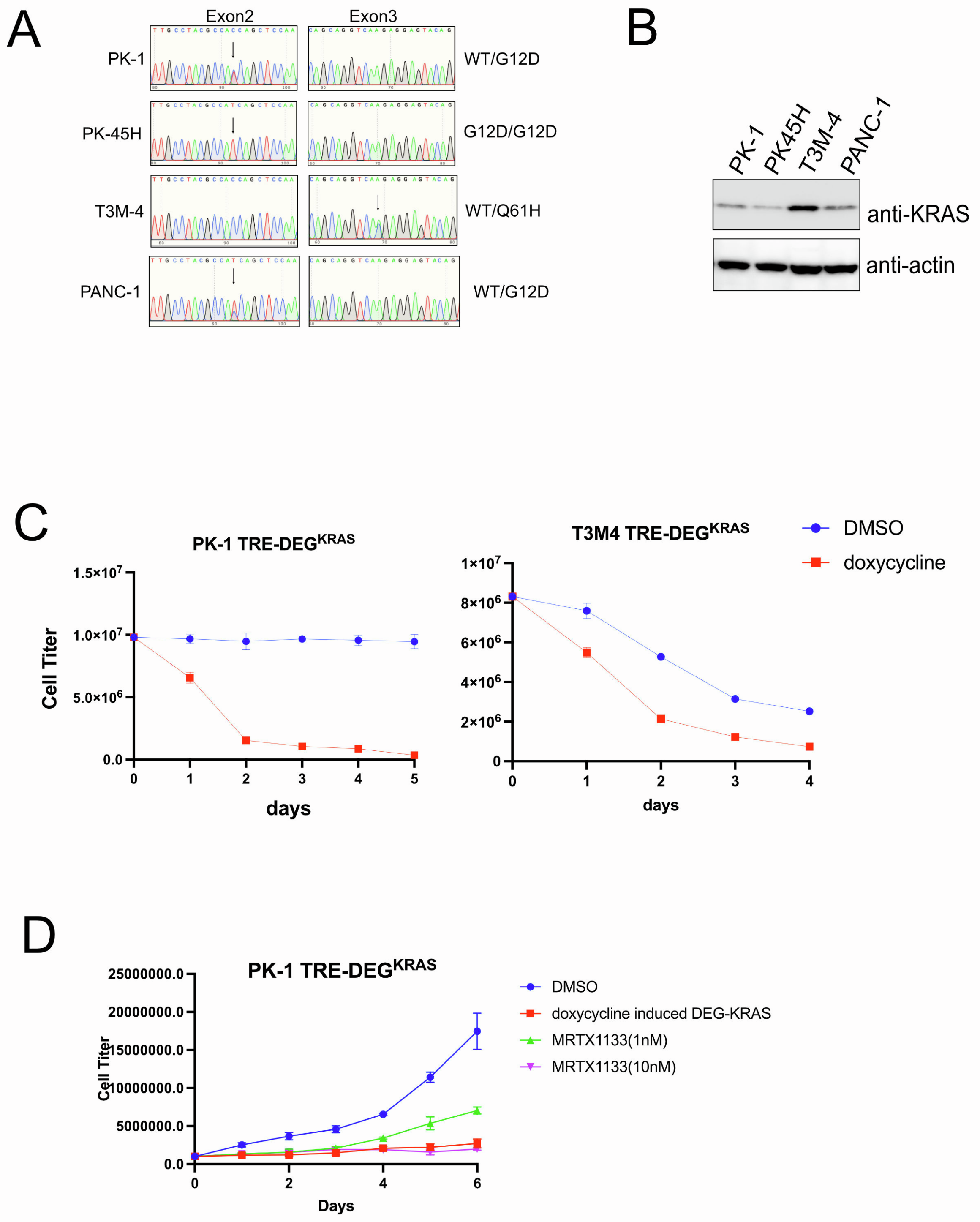
A
Exon2
Exon3
PK-1
WT/G12D
PK-45H
G12D/G12D
T3M-4
WT/Q61H
PANC-1
WT/G12D
B
PK-1
PK45H
T3M-4
PANC-1
anti-KRAS
anti-actin
C
PK-1 TRE-DEG^KRAS
Cell Titer
1.5×10^7
1.0×10^7
5.0×10^6
0.0
0
1
2
3
4
5
days
T3M4 TRE-DEG^KRAS
1×10^7
8×10^6
6×10^6
4×10^6
2×10^6
0
0
1
2
3
4
days
DMSO
doxycycline
D
PK-1 TRE-DEG^KRAS
Cell Titer
25000000.0
20000000.0
15000000.0
10000000.0
5000000.0
0.0
0
2
4
6
Days
DMSO
doxycycline induced DEG-KRAS
MRTX1133(1nM)
MRTX1133(10nM)

Figure S5. Additional characterization of KRAS dependency and DEG-KRAS effects.

(A) KRAS mutation status of five pancreatic cancer cell lines determined by Sanger sequencing.

(B) Baseline KRAS protein expression across the cell lines assessed by immunoblotting.

(C) Cell proliferation of PK-1 and T3M-4 cells expressing DEG-KRAS under suspension (non-adherent) culture conditions.

(D) Comparison of the inhibitory effects of MRTX1133 (KRAS G12D inhibitor) and doxycycline-induced DEG-KRAS expression in PK-1 cells.

Data are presented as mean ± SD of three independent biological replicates.

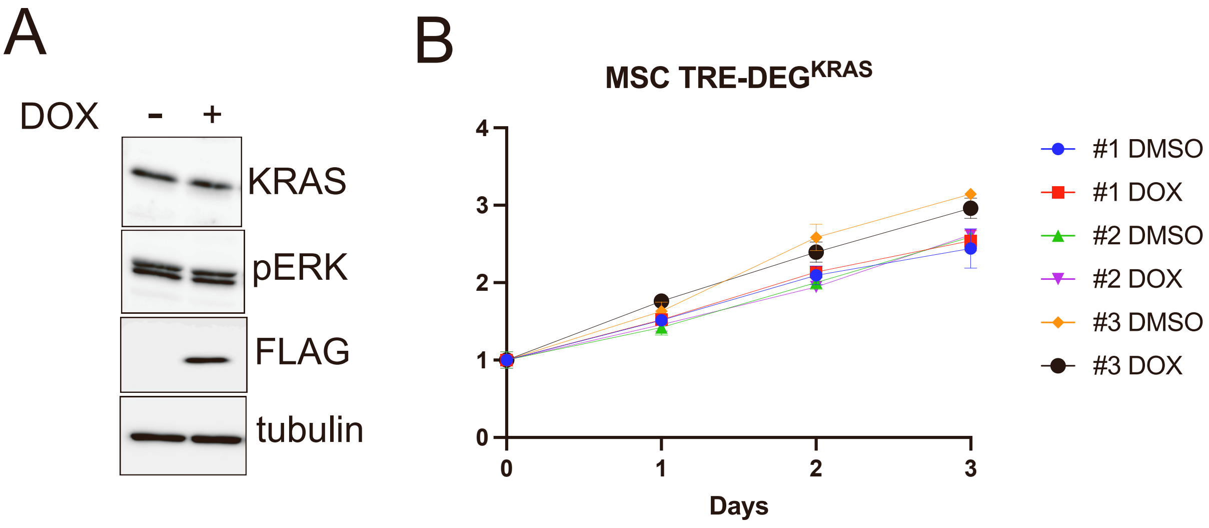

FIgure S6

(A) Western blot confirmation of DEG-KRAS expression in doxycycline-induced MSCs, but KRAS degradation or pERK suppression was not observed.

(B) Proliferation of MSCs was unaffected by DEG-KRAS expression.

Data are presented as mean ± SD of three independent biological replicates.

Table S1

Proteins tested in this paper were listed.

number	Gene	Substrate Recognision	partner Cullin	synonyms	length (a.a)
1	FBXL1	C-teminus	CUL1	SKP2	424
2	FBXO6	C-teminus	CUL1	FBX6	293
3	FBXO21	C-teminus	CUL1/CUL3	FBX21	628
4	FBXO27	C-teminus	CUL1	FBX27	283
5	FBXO41	C-teminus	CUL7	FBX41	875
6	FBW1B	C-teminus	CUL1	FBXW11	542
7	FBXW5	C-teminus	CUL7/CUL3	FBW5	566
8	FBXW9	C-teminus	CUL7	FBW9	458
9	FBXW12	C-teminus	CUL1	FBW12	464
10	WSB1	N-terminus	CUL5/CUL2	SWIP1	421
11	VHL	N-terminus	CUL2,/CUL3/CUL5	proteinG7	213
12	CRBN	C-teminus	CUL4		442
13	SOCS1	N-terminus	CUL2	SSI1, TIP3	211
14	SPOP	N-terminus	CuUL3	HIB homolog 1	374

Table S2

Antibodies used in this study were listed.

antigen	host	supplier	Cat #
GFP	rabbit	proteintech	66301-1-Ig
FLAG	mouse	Wako	014-27763
SPOT	alpaca	proteintech	ebAF488
KRAS	rabbit	thermo	12063-1-AP
ERK	mouse	SCBT	sc-514302
p-ERK	mouse	SCBT	sc-81492
HA	rabbit	proteintech	51064-2-AP
tubulin	mouse	Wako	013-25033
p-Akt	mouse	proteintech	66444-1-Ig
RhoA	mouse	SCBT	sc-418
actin	rabbit	proteintech	23660-1-AP
